# Realizing Formation and Decomposition of Li_2_O_2_ on Its Own Surface with a Highly Dispersed Catalyst for High Round-Trip Efficiency Li-O_2_ Batteries

**DOI:** 10.1016/j.isci.2019.03.013

**Published:** 2019-03-15

**Authors:** Li-Na Song, Lian-Chun Zou, Xiao-Xue Wang, Nan Luo, Ji-Jing Xu, Ji-Hong Yu

**Affiliations:** 1 College of Chemistry, State Key Laboratory of Inorganic Synthesis and Preparative Chemistry, International Center of Future Science, Jilin University, Changchun 130012, P.R. China

**Keywords:** Electrochemistry, Energy Storage, Energy Materials

## Abstract

The rapid and effective formation and decomposition of Li_2_O_2_ during cycling is crucial to solve the problems associated with the practical limitation of lithium-oxygen (Li-O_2_) batteries. In this work, a highly dispersed electrocatalyst with Ru nanoclusters inside the special organic molecular cage (RuNCs@RCC3) through a reverse double-solvent method for Li-O_2_ batteries has been proposed for the first time. This RuNCs@RCC3 shows an effective catalyst enabling reversible formation and decomposition of the Li_2_O_2_ at the interface between the Li_2_O_2_ and the liquid electrolyte, rather than the sluggish solid-solid interface reactions on commonly used solid catalysts. As a result, the Li-O_2_ cells with RuNCs@RCC3 show enhanced electrochemical performance, including low overpotential (310 mV at a current density of 100 mA g^−1^), high specific capacity (15,068 mAh g^−1^), good rate capability (1,800 mAh g^−1^ at a current density of 2.8 A g^−1^), and especially superior cycle stability up to 470 cycles.

## Highlights

• A novel highly dispersed electrocatalyst RuNCs@RCC3 for Li-O_2_ batteries was designed
• Rapid formation and decomposition of large Li_2_O_2_ on its own surface
• Excellent electrochemical performance of RuNCs@RCC3 in Li-O_2_ batteries
• Superior stability and durability of RuNCs@RCC3 in Li-O_2_ batteries

## Graphical Abstract

**Figure:**
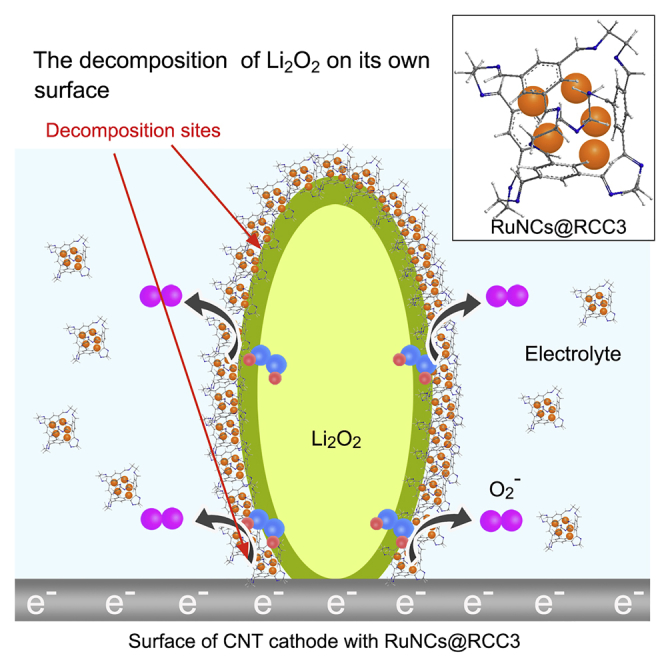

## Introduction

Rechargeable lithium-oxygen (Li-O_2_) batteries can provide a theoretical energy density of 3,600 Wh kg^−1^, delivering five times the energy density of the state-of-the-art Li-ion batteries, which are promising for electric vehicle applications (Asadi et al., 2018, Gallant et al., 2013, Xu et al., 2017). However, there are still several serious challenges for further promoting, including the slow kinetics, large overpotential, low specific capacity, poor rate capability, and cycle stability (McCloskey et al., 2013, Yu et al., 2018, Oh et al., 2012, Yao et al., 2015). The difficulty in formation and decomposition of the discharge product (Li_2_O_2_) during cycling for the Li-O_2_ system is at the heart of the problem. The specific capacity, the rate capability, the overpotential, and the cycle life are determined by the amount, the morphology, the accumulation behavior, and the formation and decomposition pathway of Li_2_O_2_, respectively. Previous studies have reported that these can be partially overcome by tailoring the nature of Li_2_O_2_. For example, Johnson et al. (2014) have proposed that high-donor-number solvents (electrolytes) can induce Li_2_O_2_ particle growth in solution, leading to sustained discharge and higher capacities. Aetukuri et al., 2015, Adams et al., 2013, and Mitchell et al. (2013) have elucidated that trace amounts of electrolyte additives (such as H_2_O and CH_3_OH), or the low current density, could facilitate the formation of Li_2_O_2_ toroids. Our previous studies (Xu et al., 2013, Xu et al., 2016) have demonstrated that use of sophisticated cathode possessing targeted properties could tailor the deposition and the morphology of Li_2_O_2_, and thus improve the electrochemical performance of Li-O_2_ batteries. Although the discharge capacity and the rate capability have been effectively improved, the slow kinetics of the large insoluble Li_2_O_2_ decomposition during charge is still a daunting challenge, and more effort is needed. Therefore various catalysts including metal oxides (Geng and Ohno, 2015, Liu et al., 2014a, Liu et al., 2014b, Yilmaz et al., 2013), metal nitrides (Shui et al., 2012, Kundu et al., 2015), metal nanoparticles (Yang et al., 2014, Lin et al., 2018a, Lin et al., 2018b), and organometallic compounds (Ren et al., 2011, Freunberger et al., 2011) have been used for the Li_2_O_2_ decomposition during charge. Even if significant progress in the overpotential of oxygen reduction reaction (ORR) and oxygen evolution reaction (OER) has been achieved, there are still some serious issues concerning the usage of those solid catalysts, which need to be resolved. The insoluble Li_2_O_2_ particles covering the solid catalysts' surface during discharging could lead to the degradation of the cathode due to the toxic effect on the catalyst. Especially, it would also cause voltage polarization and slow the electrochemical kinetics at the solid (Li_2_O_2_)-solid catalyst interface with rare reaction sites during discharge/charge (Chang et al., 2017). Studies have shown that soluble redox mediators (RMs) would be promising candidates for lowering the overpotential of ORR and OER (Sun et al., 2014, Gao et al., 2016). By tuning the formation and decomposition pathway of Li_2_O_2_ from the limited surface to the solution, the RMs significantly improve the specific capacity and reduce the overpotential of the Li-O_2_ batteries, which can be a promising strategy to realize rapid reversible cycling of Li_2_O_2_ (Chen et al., 2013). However, the RMs in Li-O_2_ batteries may have some toxic side effects, such as shuttle reactions and detrimental interactions with the Li-metal anodes of these cells. Worse still, the organic materials that are suited to serve as RMs due to C-H bonds next to O or N atoms are likely to react with the O_2_^2−^ or O_2_^−^ formed in ORR (Park et al., 2018). Therefore, the development of highly stable soluble catalysts to efficiently catalyze Li-O_2_ reactions, which simultaneously possess good inert nature toward Li anode and reduced oxygen species (Li_2_O_2_, LiO_2_), is highly desirable but still challenging. Recently, several researches are exploring to solve these questions such as using ruthenium-based catalyst (Cai et al., 2018, Lin et al., 2016) and soluble electrocatalyst (Lin et al., 2018a, Lin et al., 2018b), which exhibit excellent electrochemical performance.

With these factors in mind, a highly dispersed electrocatalyst with superior catalytically active Ru nanoclusters inside the special organic molecular cage (RuNCs@RCC3) through a reverse double-solvent method for Li-O_2_ batteries has been proposed. Also, the RuNCs@RCC3 can achieve rapid formation and decomposition (Li_2_O_2_) at the interface between Li_2_O_2_ and electrolyte. Furthermore, the as-prepared catalysts possess excellent catalytic activities, stability, and durability owing to the good confinement of Ru nanoclusters to the discrete RCC3 matrix. Consequently, the catalysts endow the batteries with outstanding performance, including relatively low charge voltage, ultrahigh specific capacity, and long cycle life.

## Results and Discussion

### Synthesis and Characterization of the RuNCs@RCC3 Catalyst

**Figure 1:**
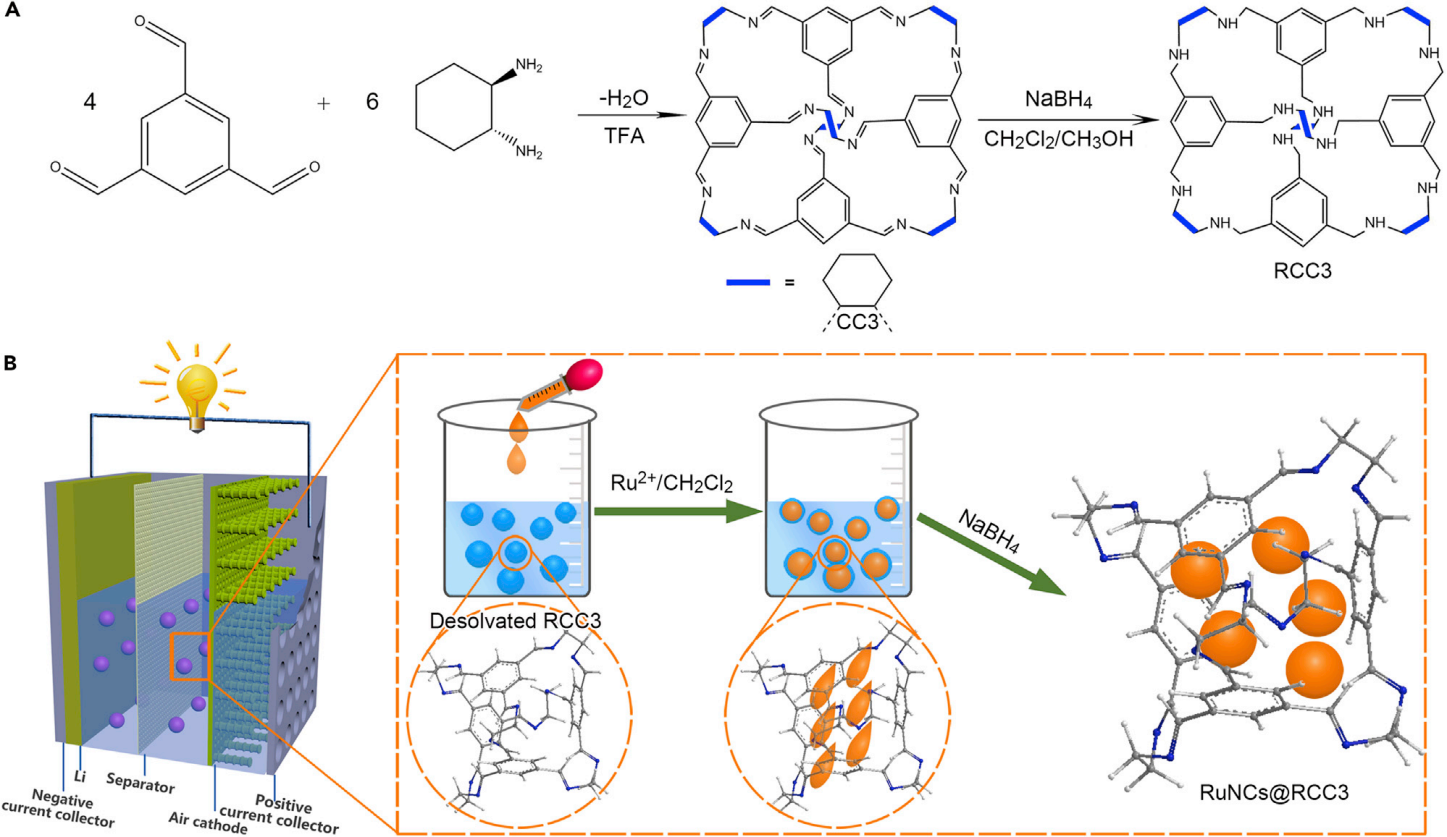
Scheme for the Fabrication of RuNCs@RCC3 (A) Synthesis of the CC3R cage by a [4 + 6] cycloimination and the reduction of CC3R to RCC3 cage by NaBH_4_. (B) Schematic illustration of the encapsulation of Ru nanoclusters inside the RCC3 matrix using a reverse double-solvent approach.

**Figure 2:**
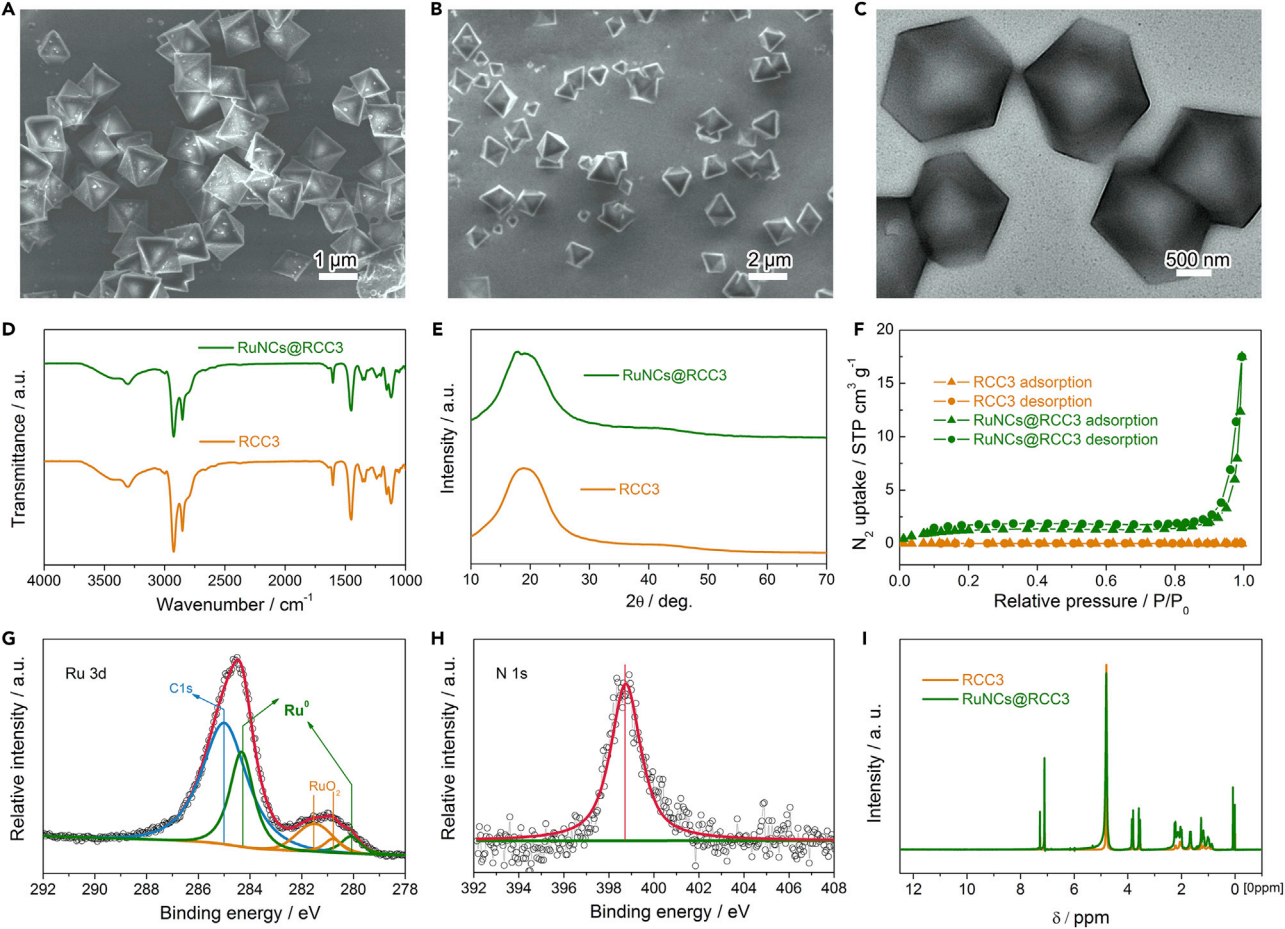
Morphological and Structural Characterization of RuNCs@RCC3 (A) FESEM image of RCC3. See also Figure S7. (B and C) Scanning electron microscopic (B) and TEM (C) images of RuNCs@RCC3. See also Figures S8 and S9. Scale bars, 1 μm in (A), 2 μm in (B) and 500 nm in (C). (D–F) FTIR patterns (D), PXRD patterns (E), and N_2_ adsorption-desorption isotherms (F) of RCC3 and RuNCs@RCC3. See also Figures S2 and S10. (G and H) Ru 3d XPS spectrum (G) and N 1s XPS spectrum (H) of the RuNCs@RCC3. (I) ^1^H NMR spectra of RCC3 and RuNCs@RCC3. See also Figure S4.

The synthetic process and mechanism of encapsulating Ru nanoclusters (RuNCs) inside RCC3 catalysts (RuNCs@RCC3) are illustrated in Figure 1. To design a highly dispersed catalyst, the amine cage RCC3 was selected as the support because of its excellent solubility in electrolyte solvents (Hasell and Cooper, 2016, Yang et al., 2018). As shown in Figure 1A, the chiral imine cage CC3R was formed by cycloimination of 1,3,5-triformylbenzene and (1R,2R)-1,2-diaminocyclohexane and exhibited an apparent Brunauer-Emmett-Teller surface area of ∼500 m^2^ g^−1^ in highly crystalline form (Figure S1). Then RCC3 can be easily synthesized by reducing CC3R to the corresponding dodecaamine cage RCC3 using NaBH_4_ with an yield close to 100% (Briggs and Cooper, 2017), which is further confirmed by Fourier transform infrared spectroscopy (FTIR, Figures S2 and 2D), ^1^H and ^13^C nuclear magnetic resonance (NMR) spectroscopy (Figures 2I, S3, and S4), mass spectrum (Figure S5), and elemental analysis (Table S1) (Liu et al., 2014a, Liu et al., 2014b). Figure 1B shows the synthesis and working mechanism of the RuNCs@RCC3 for Li-O_2_ batteries. CH_2_Cl_2_ molecules were encapsulated in RCC3 cages by the reverse double-solvent approach to disperse the RuNCs@RCC3 in the electrolyte. A small amount of Ru(C_5_H_7_O_2_)_3_/CH_2_Cl_2_ as hydrophobic solution was slowly added into the organic cage/water dispersion; subsequently, the as-prepared NaBH_4_ aqueous solution was employed to rapidly reduce the metal precursors; and the RuNCs@RCC3 was successfully prepared. The corresponding powder X-ray diffraction (PXRD) measurement is displayed in Figure S6.

The morphology of the RuNCs@RCC3 was investigated by field emission scanning electron microscopy (FESEM) and transmission electron microscopy (TEM). As shown in Figures 2A and S7, the stable RCC3 presents octahedron crystals with the size of ∼2 μm. Similar to the RCC3, no changes in structure and composition were observed in the FESEM (Figure 2B) and TEM (Figure 2C) images and the FTIR spectra (Figure 2D) of the as-prepared RuNCs@RCC3, suggesting that the RCC3 is intact after the encapsulation of RuNCs. The PXRD pattern of RuNCs@RCC3 shows a broad peak in the range of 10°–30°, further suggesting that the RCC3 cage is well maintained (Figure 2E). Meanwhile, no diffraction peaks of RuNCs are observed, indicating the successful formation of ultra-small well-dispersed RuNCs. As described in the N_2_ adsorption-desorption isotherms (Figure 2F), desolvated RCC3 shows no porosity to N_2_ owing to its collapsed structure, and as revealed in the Figure S10, the Ru nanoparticles (RuNPs)/RCC3 shows a rare nonporous characteristic, which should be ascribed to the aggregation of RuNPs on the surface of RCC3. On the contrary, the obtained RuNCs@RCC3 achieves an increased porosity to N_2_, illustrating that the incorporation of RuNCs inside the pores of RCC3 contributes to the increased porosity of RCC3. In addition, X-ray photoelectron spectroscopy (XPS) was further adopted to identify the nature of the RuNCs@RCC3 surface. As shown in Figure 2G, the binding energies at 280.2 and 284.3 eV are attributed to Ru 3d_5/2_ and Ru 3d_1/2_, respectively (Chakroune and Viau, 2005, Hrbek, 1986). The binding energies at 280.9 and 281.5 eV are ascribed to the trace amounts of RuO_2_, which originated from the surface oxidation of Ru in air. In addition, the peak at 284.6 eV belongs to the main C-C bond (Figure 2G). The N1s XPS spectrum shows the major existence of pyrrodic N at 398.8 eV in Figure 2H. However, the RuNCs can hardly be observed through the TEM of RuNCs@RCC3 (Figure S8) due to their ultrafine size, which is consistent with the result of energy-dispersive X-ray spectroscopy elemental mapping (Figure S9), indicating that the RuNCs were successfully embedded in the cage cavities. NMR measurement was used to study the structure of RuNCs@RCC3, particularly the spatial relationship between RCC3 and the encapsulated RuNCs (Victoria et al., 2009). The ^13^C NMR spectrum of RuNCs@RCC3 is identical to that of RCC3 (Figure S4), demonstrating the stable configuration of the RCC3 cages. On the contrary, all the peaks in the ^1^H NMR spectrum of RuNCs@RCC3 (Figure 2I) are broadened compared with those of RCC3 due to the encapsulation of RuNCs. The mass spectrum of the RuNCs is not obtained because of the low intensities of the expected large number of isotope peaks, owing to the broad and continuous small particle size distribution (Figure S5). These results further prove the successful entering of RuNCs into the cage cavities.

### Electrochemical Properties and Discharging Mechanism of the RuNCs@RCC3 in Li-O_2_ Cells

**Figure 3:**
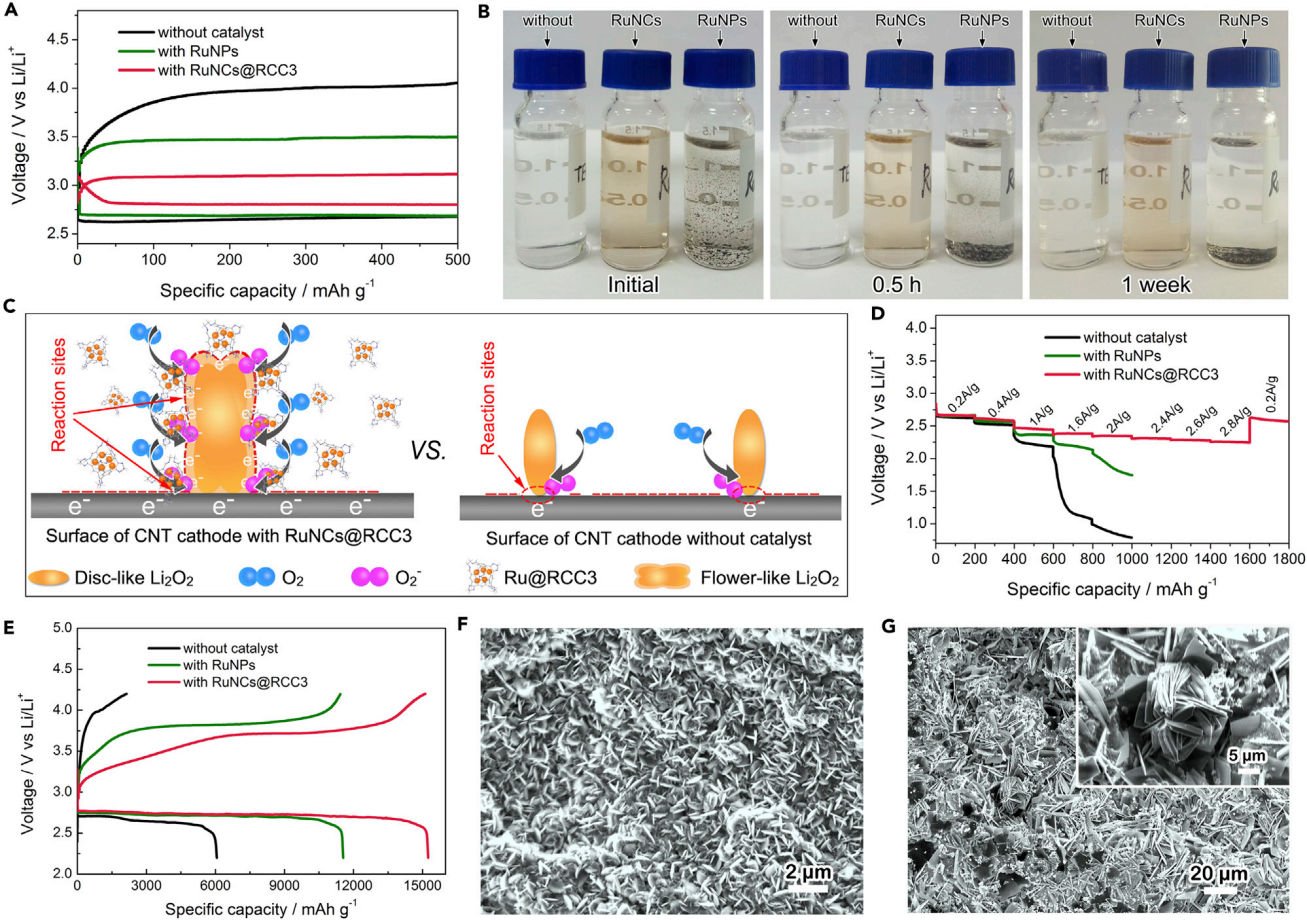
Electrochemical Performance and Characterization of Discharged Products (A) First charge-discharge curves of lithium-oxygen (Li-O_2_) cells at a current density of 100 mA g^−1^, and a specific capacity limit of 500 mAh g^−1^. (B) Photographs of the pristine electrolyte (left), RuNCs@RCC3 (middle), and RuNPs/RCC3 (right) catalysts in CH_2_Cl_2_/TEGDME (v/v, 1/2) at different stages (C_m_ = 6.67 mg/mL). (C) An electrochemical mechanism for the aggregation of Li_2_O_2_ on the surface of the CNT with RuNCs@RCC3, versus without catalyst. (D and E) The rate capability (D) of the Li-O_2_ cells with three types of catalysts at different current densities. Galvanostatic discharge and recharge curves (E) of the Li-O_2_ cells with three kinds of catalysts at a current density of 100 mA g^−1^. See also Figures S12 and S13. (F and G) FESEM image of the discharged CNT cathode without catalyst (F) and with RuNCs@RCC3 (G) at a current density of 200 mA g^−1^ and a specific capacity of 2,000 mAh g^−1^. The inset of (G) represents the corresponding enlarged FESEM image. See also Figure S14.

Li-O_2_ batteries with a carbon nanotube (CNT) (Figure S11) cathode, a lithium anode, and tetraethylene glycol dimethyl ether (TEGDME) and CH_2_Cl_2_ (V_TEGDME_:V_CH2Cl2_ = 2:1)/LiTFSI electrolyte were assembled to further study the formation and decomposition of Li_2_O_2_ during cycling. All results for the specific capacities and current densities are calculated based on the total carbon mass on the cathode (0.45 mg cm^−2^). The electrochemical behavior of Li-O_2_ cell is shown in Figure 3. For comparison, the cells without catalyst and with RuNPs were assembled respectively. Figure 3A displays the first discharge-charge voltage profiles of the Li-O_2_ cells with RuNCs@RCC3, RuNPs, and without catalyst at a current density of 100 mA g^−1^. The discharge and charge voltages of the Li-O_2_ cells can be improved with the RuNCs@RCC3 catalysts, leading to the enhancement the round-trip efficiency, which is vital for energy storage devices. The discharge voltage of the cell with RuNCs@RCC3 is obviously higher than that of RuNPs by ∼123 mV and without catalyst by ∼178 mV. However, the charge voltage with RuNCs@RCC3 is much lower than that with RuNPs by ∼383 mV and without catalyst by ∼920 mV, which is further supported by the excellent solubility of RuNCs@RCC3 in the electrolyte. The solubility tests of TEGDME, RuNCs@RCC3, and RuNPs/RCC3 in CH_2_Cl_2_/TEGDME at different stages are displayed in Figure 3B. Interestingly, RuNCs@RCC3 can be highly dispersed to form a transparent solution and remains unchanged even after a week. However, the as-prepared RuNPs/RCC3 catalyst is insoluble as a dark dispersion. Presumably, the RCC3 cage shell serves as an effective protector for Ru nanoclusters, preventing them from aggregations, as well as endowing them with a high dispersibility and more active sites. The excellent solubility of RuNCs@RCC3 in the electrolyte offered more exposed reaction sites for the electrochemical reactions on the cathode and Li_2_O_2_ surface (Figure 3C). On the contrary, only a small number of reaction sites existed on the cathode surface with RuNPs or without catalyst, showing poor electrochemical performance toward both formation and decomposition of Li_2_O_2_ (Figure 3C). Furthermore, benefitting from the RuNCs@RCC3, the cells exhibit high rate capacity. The discharge voltage plateau of the cells with RuNCs@RCC3 was higher than that with RuNPs or without catalyst at different current densities (Figure 3D), which is consistent with the discharge-charge voltage profiles in Figure 3A. The electrochemical impedance spectra of these three types of Li-O_2_ cells were displayed in Figure S12. The interfacial resistance of the cell with RuNCs@RCC3 is lower than that with RuNPs or without catalyst, demonstrating the better rapid electron, ionic, and mass transport, as well as the improved rate capability. Furthermore, Figure 3E shows that RuNCs@RCC3 delivers a much higher discharge capacity of 15,068 mAh g^−1^ than the cells with RuNPs and without catalyst (11,536 and 6,066 mAh g^−1^, respectively). Meanwhile, the charge capacity for Li-O_2_ cell with RuNCs@RCC3 below 4.2 V is close to the discharge capacity, whereas the coulombic efficiency of the cell without catalyst is only ca. 35.2%, demonstrating a significantly enhanced charging efficiency. What's more, even at a very high current density of 1,000 mA g^−1^, the discharge capacity can still reach 2,893 mAh g^−1^ (Figure S13). The difference in the specific capacities of the cell with RuNCs@RCC3 or without catalyst might be ascribed to the different deposition behaviors and morphologies of the discharge product. And for this, the morphologies of the discharge product on the two cathodes were studied. The related FESEM images of the two discharged cathodes at different capacities of 500 and 2,000 mAh g^−1^ are displayed in Figures S14, 3F, and 3G. The discharged CNT cathode without RuNCs@RCC3 catalyst shows small disks/toroid (100–200 nm in size) morphology of the Li_2_O_2_ at a specific capacity of 500 mAh g^−1^ (Figure S14A). With the increasing discharge capacity, the cathode surface is almost fully covered by the small disk-like Li_2_O_2_ particles (∼200nm in size) (Figure 3F), which would execrably impede Li^+^, O_2_, and charge transfer on the cathode during subsequent discharge, resulting in severe polarization and premature finish of the discharge (Gallant et al., 2013). By contrast, on the surface of the cathode with RuNCs@RCC3, the aggregated micrometer-sized flower-like products could be clearly observed (Figure S14B, 2 μm in size, and Figure 3G, 10–20 μm in size). This obvious difference can be ascribed to the growth pathway of Li_2_O_2_ with RuNCs@RCC3 possessing more reaction sites, which can induce more LiO_2_* nucleation to generate large Li_2_O_2_, which is beneficial to maintain the active sites for ORR, and enable a high discharge specific capacity of the Li-O_2_ cell. To definitively demonstrate the effect of the catalyst, rather than other influences such as the excess water in the electrolyte, Karl Fischer titration on the neat electrolyte, the electrolytes after the addition of RuNCs@RCC3, and the electrolyte after cell cycling was carried out (Figure S15). The results demonstrate that the water content in the electrolyte shows slight increment with the addition of RuNCs@RCC3. Even after the 20th cycle, the water content in the electrolyte with RuNCs@RCC3 is still lower than 50 ppm. Therefore, the formation of large Li_2_O_2_ is due to the RuNCs@RCC3 and not because of excess water in the electrolytes. Figure 3C shows the electrochemical growth mechanism of the disk-like and flower-like Li_2_O_2_. The ORR reaction only occurred on the surface of the cathode without RuNCs@RCC3 limited by the distribution of the active sites. As the cathode surface would gradually be covered by the insoluble Li_2_O_2_ particles with the continuous discharge, it is difficult to obtain the larger Li_2_O_2_, resulting in premature finish of the discharge process. In sharp contrast, on the cathode with RuNCs@RCC3, the ORR reaction can occur on the surface of the discharge product Li_2_O_2_, resulting in the continuous growth of Li_2_O_2_ and the eventual formation of micrometer-sized flower-like assemblies. In detail, high solubility and accessibility of the open “skeleton” create a fast pathway for highly active RuNCs “blood” to reach the surface of Li_2_O_2_ (Viswanathan et al., 2011): the enhanced conductivity of the outer non-stoichiometric Li_2_O_2_ by deposited RuNCs enables electrons to be transferred to the surface of Li_2_O_2_, which ensures all of the conditions for ORR reaction. As shown in Figure S16, the XRD patterns of the discharge cathodes prove that Li_2_O_2_ is the only crystalline product in the two cases, despite the different morphologies of the discharged product. In addition, the peak of the LiOH can hardly be observed around 2-theta angles of 20° (Figure S17), also indicating that Li_2_O_2_ is the only crystalline product. According to the titration experiment (Figure S18), the yield of Li_2_O_2_ on the surface of CNT cathodes with RuNCs@RCC3 after the first discharge to 1.5 mAh was found to be about 73.5% compared with the theoretical capacity at the current density of 200 mA g^−1^, further confirming that the discharge capacity of the CNT cathode with RuNCs@RCC3 catalysts is mainly due to the formation of Li_2_O_2_.

### Reversibility and Charging Mechanism of the RuNCs@RCC3 in Li-O_2_ Cells

**Figure 4:**
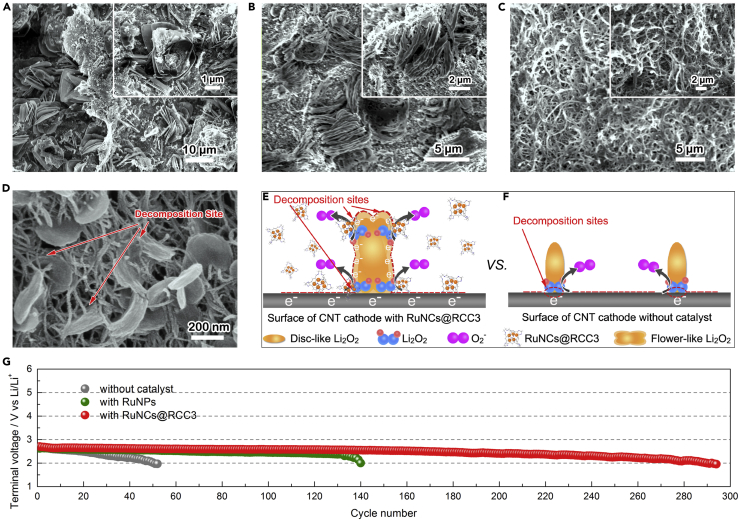
Cycle Stability and Characterization of Charged Products (A–C) FESEM images of the recharged CNT cathode with RuNCs@RCC3 at a current density of 200 mA g^−1^ and charge capacities of 500 (A), 1,000 (B), and 2,000 mAh g^−1^ (C). Insets in (A–C) show the corresponding enlarged FESEM images. (D) FESEM image of the recharged CNT cathode with RuNPs at a current density of 200 mA g^−1^ with a charge capacity of 1,000 mAh g^−1^. (E and F) Schematic of the Li_2_O_2_ oxidation mechanism in electrolyte with RuNCs@RCC3 (E) and without catalyst (F). (G) Variation of the terminal voltage upon the discharge of the Li-O_2_ cells at a current density of 200 mA g^−1^ and a specific capacity limit of 500 mAh g^−1^ with three types of catalysts. See also Figure S19.

The mechanism of the Li_2_O_2_ decomposition process was systematically investigated by FESEM during recharging in Figure 4. Large flower-like Li_2_O_2_ was formed on the surface of the CNT cathode with RuNCs@RCC3 (Figure 3G) at a capacity of 2,000 mAh g^−1^ and immediately begins to melt when recharging to 500 mAh g^−1^ (Figure 4A); the partial flower-like Li_2_O_2_ crystals remain visible through the FESEM micrograph. The Li_2_O_2_ crystals gradually decrease in size and disappear at the end of the recharging, which is described by the FESEM images at capacities of 1,000 mAh g^−1^ (Figure 4B) and 2,000 mAh g^−1^ (Figure 4C). Figure 4E shows the Li_2_O_2_ decomposition process affected by the RuNCs@RCC3 catalyst. For the cathode with the RuNCs@RCC3, the decomposition sites were exposed both on the surface of the cathode and the flower-like Li_2_O_2_. Owing to the existence of plenty of decomposition sites with RuNCs@RCC3, the flower-like Li_2_O_2_ could be easily decomposed on the interface between the solid Li_2_O_2_ and the liquid electrolyte, which is completely different from the solid-solid interface reaction catalyzed by solid catalysts (Feng et al., 2015). In contrast, we found that the electron transfer merely happens at the interface of the discoid Li_2_O_2_ and the CNT surface (Figures 4D and 4F). This traditional catalytic mechanism shows poor electrochemical kinetics on charging, leading to higher charging voltage due to the small contact area between the insoluble Li_2_O_2_ particles and the solid catalyst (consistent with Figure 3A). Based on the above results, the RuNCs@RCC3 catalyst exhibits excellent superiority in terms of the improved kinetics and thermodynamics of Li_2_O_2_ formation and decomposition, stimulating us to investigate the cycling stability of the Li-O_2_ batteries with RuNCs@RCC3. The battery tests were carried out according to the widely used capacity-limited cycle method. Surprisingly, the voltage obtained at the discharge terminal of the Li-O_2_ cells with RuNCs@RCC3 is >2 V for 470 cycles; in contrast, the discharge voltages of the RuNPs and pristine CNT are down to <2 V after 315 and 288 cycles (Figure 4G). Even with a specific capacity of 5,000 mAh g^−1^ at a current density 500 mA g^−1^, the Li-O_2_ cells with RuNCs@RCC3 remain more than 14 cycles (Figure S19). These results demonstrate the excellent reversibility and cycle stability of the CNT cathode with the RuNCs@RCC3.

### Stability of the RuNCs@RCC3 in Li-O_2_ Cells

**Figure 5:**
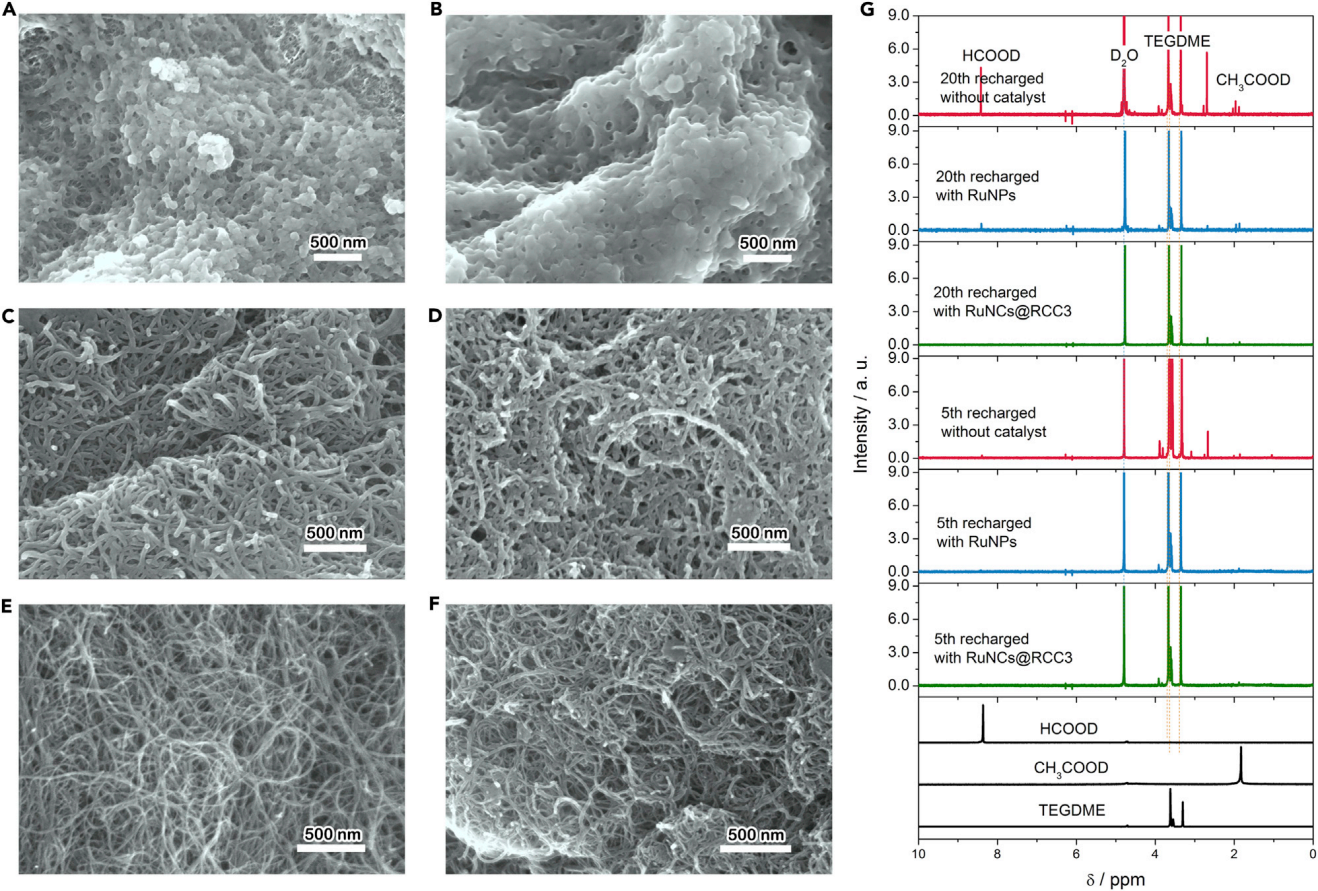
Cathode Morphology upon Cycling (A–F) FESEM images of the recharged CNT cathode without catalyst at a current density of 200 mA g^−1^ and a charge capacity of 1,000 mAh g^−1^ after the fifth recharging (A) and the 20th recharge (B). FESEM images of the recharged CNT cathode with RuNPs at a current density of 200 mA g^−1^ and a charge of 1,000 mAh g^−1^ after the fifth(C) and the 20th recharge (D). FESEM images of the recharged CNT cathode with RuNCs@RCC3 at a current density of 200 mA g^−1^ and a charge capacity of 1,000 mAh g^−1^ after the fifth recharge (E) and the 20th recharge (F). (G) ^1^H NMR spectra of the CNT cathodes without catalyst or with RuNCs@RCC3 after the fifth and 20th recharge. The spectra for TEGDME, HCO_2_Li, and CH_2_CO_2_Li are also shown for reference. See also Figure S20.

The stability of the catalyst upon cycling was further studied. The evolution of the morphology of the recharged cathode after the fifth and 20th cycles was examined (Figure 5). As shown in Figures 5E and 5F, the flower-like discharge products disappear after recharging and the clean surface of the whole cathode is almost fully recovered after the 20th recharge, indicating good reversibility and stability of the Li-O_2_ cell with RuNCs@RCC3, whereas the thin layers with muddy parasitic products appear on the cathode surface without RuNCs@RCC3 after the fifth recharging and the thickness increases with the cycling going on (Figures 5A–5D). ^1^H NMR spectra show that parasitic products are lithium formate (HCO_2_Li) and lithium acetate (CH_3_CO_2_Li) from the side reactions between the electrolyte and Li_2_O_2_ or intermediates during cycling (Figures 5G and S20). This continuous accumulation of by-products poisons the cathode surface and hinders the transmission of the intermediate, electrons, O_2_, and Li^+^ within the cathode, consequently leading to the death of the Li-O_2_ cell without RuNCs@RCC3. Also, the cathode with RuNCs@RCC3 shows much less parasitic discharged products than the other two cathodes without RuNCs@RCC3 after 5 and 20 cycles. In addition, FTIR spectra (Figure S21) demonstrate that the amount of the irreversibly decomposed product deposited on the cathode with RuNCs@RCC3 is less than that on the cathode without RuNCs@RCC3 after the 20th recharging, which is consistent with the above NMR results. These results clearly illustrate the superior electrochemical stability of the RuNCs@RCC3, which may be ascribed to its inertness toward the Li metal anode and reduced oxygen species (O_2_^2−^ or O_2_^-^) (McCloskey et al., 2013). To support this hypothesis, the RuNCs@RCC3 was physically mixed with Li metal, Li_2_O_2_, and KO_2_ in the DMSO/dicyclohexyl-18-crown-6 (crown ether) for more than 30 days, forming the metastable solvated LiO_2_ (Black et al., 2012). As shown in Figure S22, no significant change is observed in FTIR spectra after processing, indicating excellent durability and chemical stability of the RuNCs@RCC3.

**Figure 6:**
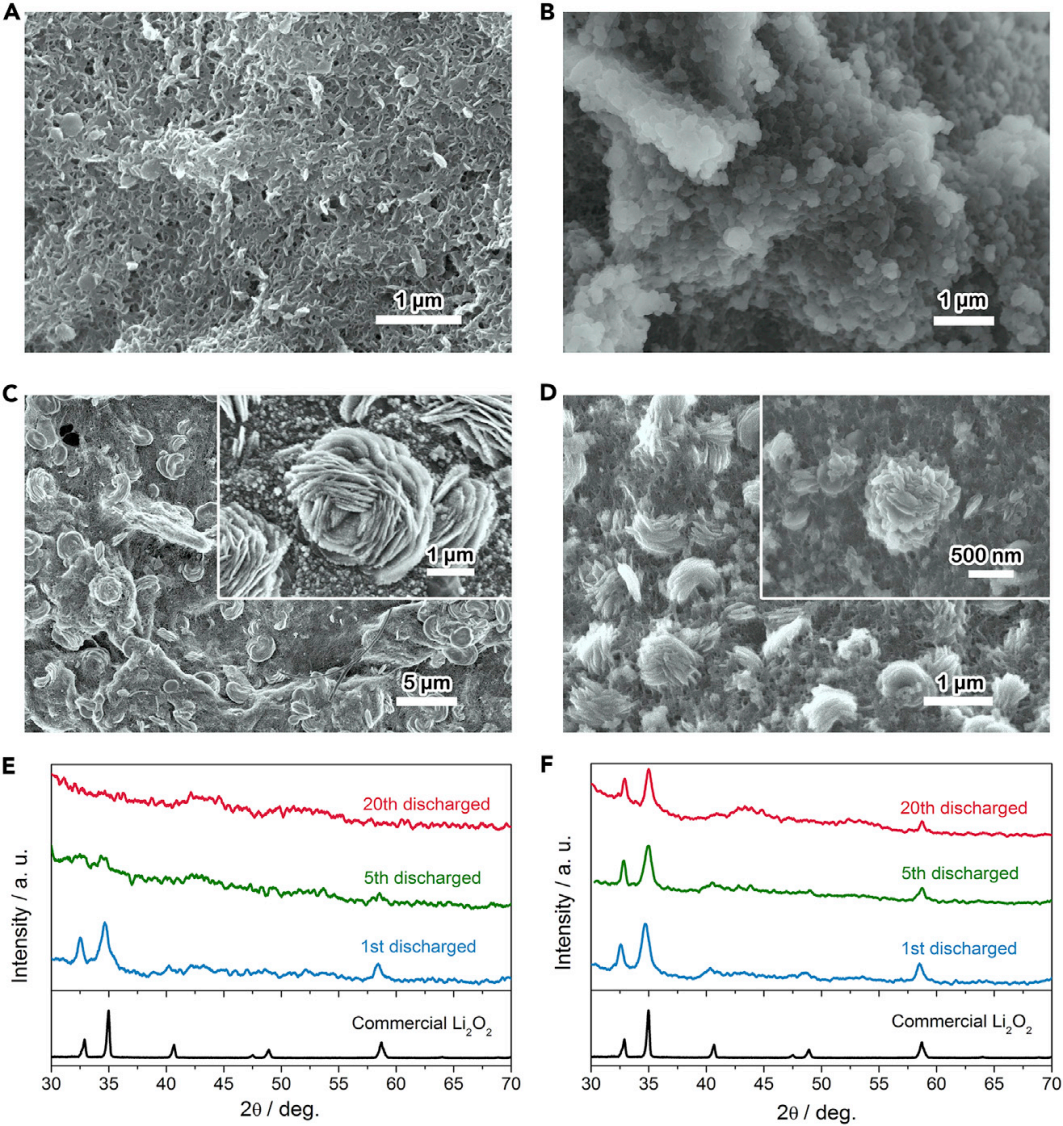
The Morphology and Crystallinity of the Discharged Product upon Cycling (A–D) FESEM images of the fifth (A) and the 20th (B) discharged CNT cathodes without catalyst at a current density of 200 mAh g^−1^ and a specific capacity of 1,000 mAh g^−1^. FESEM images of the fifth (C) and the 20th (D) discharged CNT cathodes with RuNCs@RCC3 at a current density of 200 mA g^−1^ and a specific capacity of 1,000 mAh g^−1^. Insets in (C and D) show the corresponding enlarged FESEM images. (E and F) PXRD patterns of the discharge products on the CNT cathodes without catalyst (E) and with RuNCs@RCC3 (F). See also Figure S17.

Considering the superior electrochemical stability of the RuNCs@RCC3, the evolution of the morphology and crystallinity of the discharge products after the fifth and 20th cycles was also examined. Although the discharged products are mainly the disk-like Li_2_O_2_ on the cathode without RuNCs@RCC3 after the first discharge (Figures S14A and 3F), crystalline Li_2_O_2_ becomes rarely visible at the fifth cycle and disappears after the 20th cycle (Figures 6A and 6B). The degradation of the crystallinity of Li_2_O_2_ can be ascribed to the increasing accumulation of the by-products on the cathode surface and the presence of CO_2_ in the electrolyte, which hinder the nucleation and recrystallization of Li_2_O_2_, leading to the formation of amorphous Li_2_O_2_ (Xu et al., 2016). The micrometer-sized, flower-like products can be clearly observed on the cathode with RuNCs@RCC3 even after 20 cycles (Figures 6C and 6D). The differences in the stability of Li_2_O_2_ on cycling indicate that the RuNCs@RCC3 could suppress the formation of the parasitic products during cycling, resulting in favorable rechargeability and good stability of Li-O_2_ batteries.

### Conclusions

In summary, a highly dispersed RuNCs@RCC3 catalyst realizing the formation and decomposition from the top of the large Li_2_O_2_ in Li-O_2_ cells by a reverse double-solvent approach is fabricated. The Li-O_2_ batteries with RuNCs@RCC3 are capable of low discharge-charge gap (310 mV at a current density of 100 mA g^−1^), high specific capacity (15,068 mAh g^−1^ at a current density of 100 mA g^−1^), high-rate capability (1,800 mA g^−1^ with the upper limit current density of 2.8 A g^−1^), and long-term stability (470 cycles at a current density of 200 mA g^−1^). The improved performance was attributed to the stability and durability of the catalyst with highly catalytic activity, which is vital for electrochemical catalytic reactions, including a high fraction of exposed active reaction sites, porous structure for the rapid formation and decomposition of large Li_2_O_2_, and a soluble, stable, and durable conductive network with good electroconductivity. Furthermore, the role of the RuNCs@RCC3 as an electrocatalyst for electron transfer in the electrolyte (which differs from an RM) must also be considered. The aforementioned experimental study has opened the way to further research in such highly dispersed catalysts for Li-O_2_ batteries to realize practical devices.

### Limitations of the Study

The parasitic reactions with RuNCs@RCC3 catalysts, RuNPs, and without catalyst are analyzed in detail, but the differential electrochemical mass spectroscopy (DEMS) is not available for quantitative analysis of the by-products because of the limited laboratory conditions.

## Methods

All methods can be found in the accompanying Transparent Methods supplemental file.

## Supplemental Information

Document S1. Transparent Methods, Figure S1–S22, and Tables S1 and S2
