# Supplementary material for: Realizing Formation and Decomposition of Li2O2 on Its Own Surface with a Highly Dispersed Catalyst for High Round-Trip Efficiency Li-O2 Batteries
Source: iScience. 2019 Mar 15;14:36–46. doi: 10.1016/j.isci.2019.03.013 (PMC6439306; doi:10.1016/j.isci.2019.03.013)
Supplement: Document S1. Transparent Methods, Figure S1–S22, and Tables S1 and S2 [file mmc1.pdf]

ISCI, Volume *14*

Supplemental Information

Realizing Formation and Decomposition of Li_2O_2 on Its Own Surface with a Highly Dispersed Catalyst for High Round-Trip Efficiency Li-O_2 Batteries

Li-Na Song, Lian-Chun Zou, Xiao-Xue Wang, Nan Luo, Ji-Jing Xu, and Ji-Hong Yu

Supplemental Figures

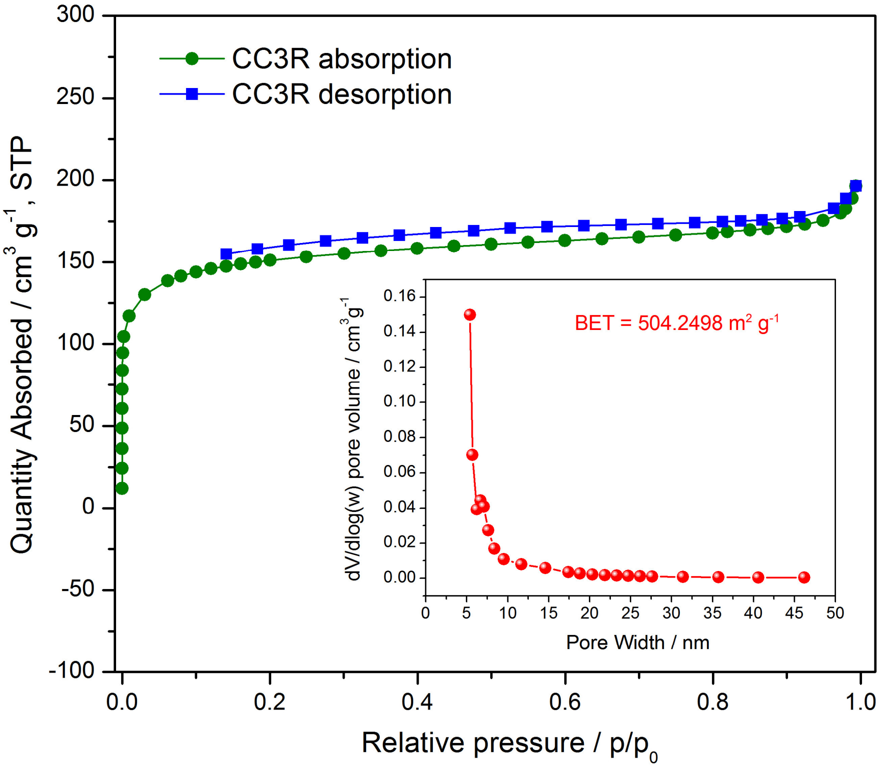

Figure S1. N_2 adsorption/desorption isotherms of CC3R. Porous organic cages are a unique class of microporous material composed of discrete molecules with intrinsic, guest accessible cavities (Briggs et al., 2017; Hasell et al., 2016; Liu et al., 2014). Supplemental Figure 1 shows the chiral imine cage with an apparent Brunauer−Emmett−Teller (BET) surface area of ~500 m^2/g, as the reduced derivative parent of RCC3 and CC3R, is formed by [4+6] cycloimination. Related to Figure 1.

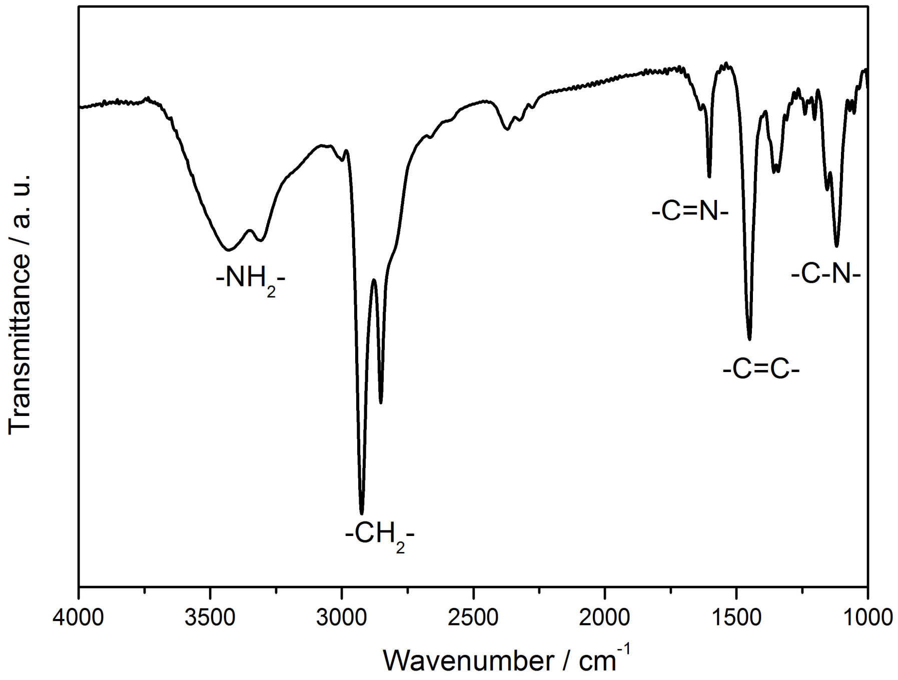

Figure S2. FTIR spectra of RCC3 (without purification). FTIR measurement shows the main peaks of -CH_2-, -C=N-, -C=C-, -C-N- in the RCC3. The double peaks near the 3400 cm^{-1} may be contributed to –NH_2- originating from the reactant of (1R,2R)-1,2-diaminocyclohexane. Related to Figure 1.

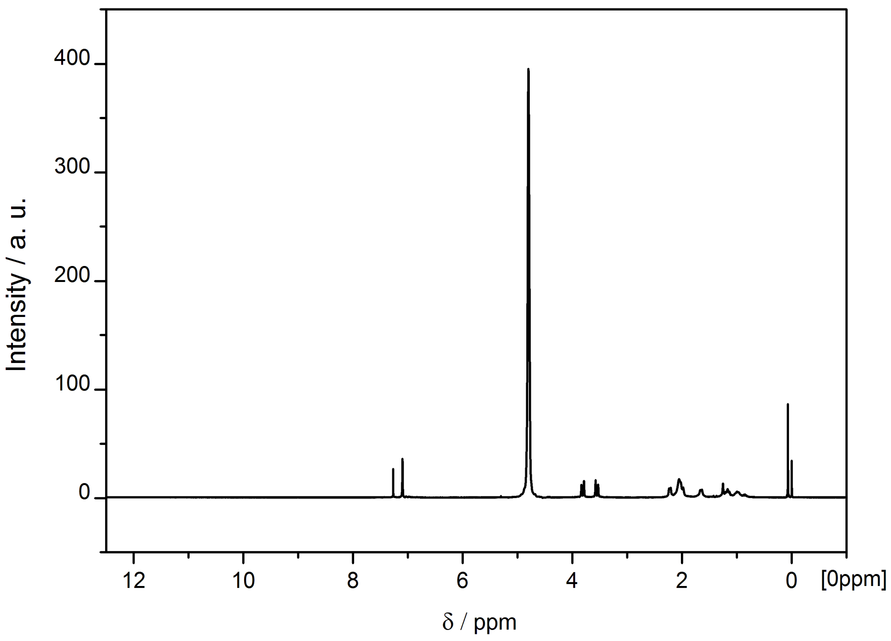

Figure S3. ^{1}H NMR spectrum of RCC3 (without purification). ^{1}H NMR (300 MHz, Chloroform-d) δ 7.31 (s, 1H), 7.13 (s,5H), 3.84 (d, J = 14.0 Hz, 6H), 3.57 (d, J = 14.1 Hz, 6H), 2.02 (d, J = 12.4 Hz, 7H), 1.66 (d, J = 8.8 Hz, 6H), 1.30 – 1.11 (m, 11H), 1.00 (s, 4H), 0.08 (s, 4H). Related to Figure 1.

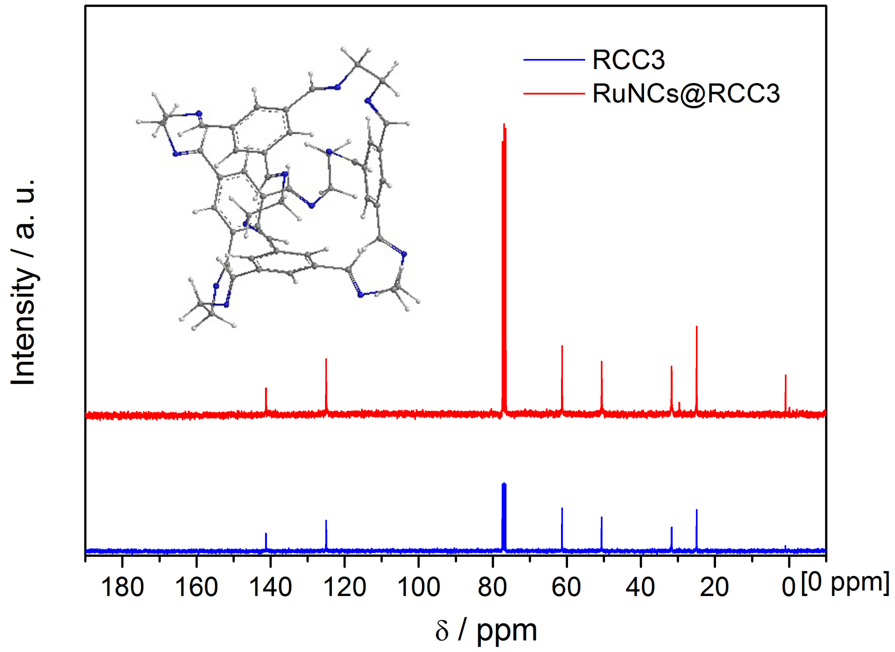

Figure S4. ^{13}C NMR spectra of RCC3, RuNCs@RCC3. The peaks of RuNCs@RCC3 are consistent with the RCC3, indicating that the encapsulation of RuNPs has no effect on the structure of RCC3. ^{13}C NMR (75 MHz, Chloroform-d) δ 141.09, 124.98, 61.18, 50.45, 31.56, 24.87. Related to Figures 1 and 2.

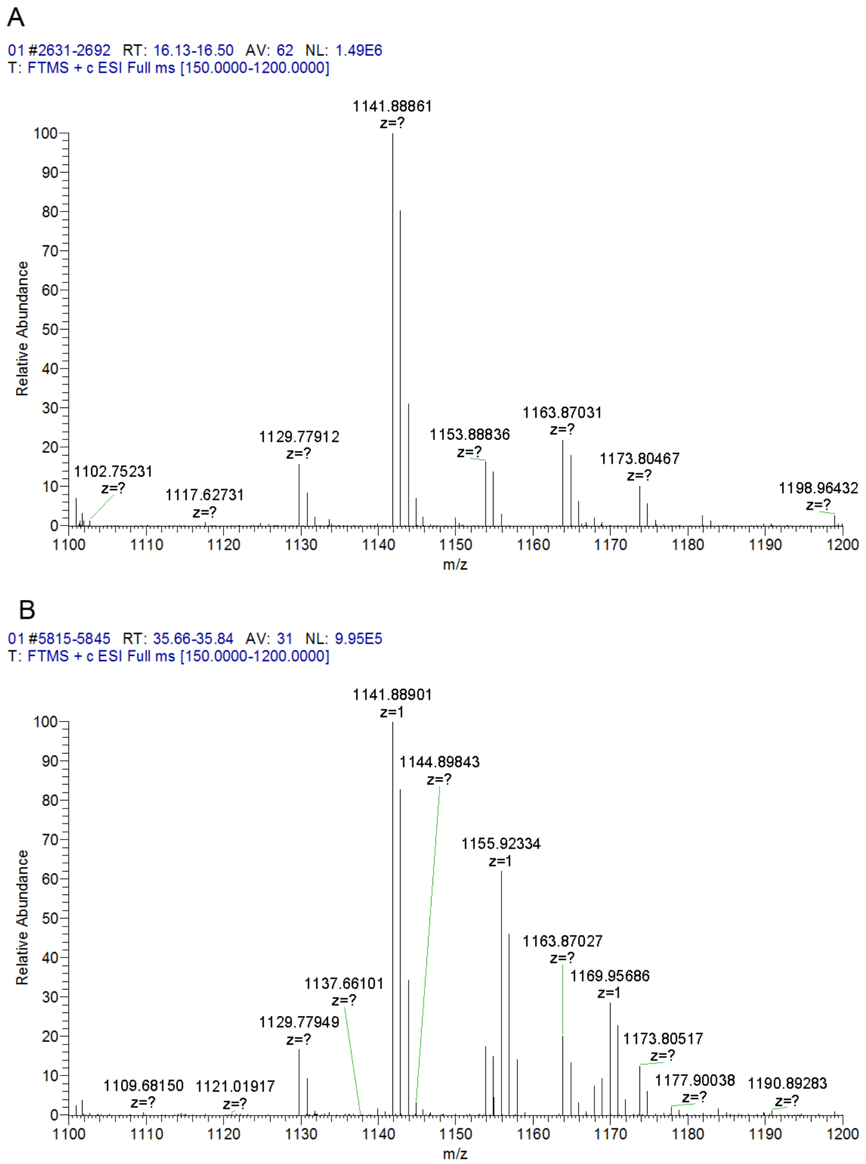

Figure S5. (**A**) FTMs + c ESI spectra of the dried RCC3 (with purification) and (**B**) RuNCs@RCC3. The molecular mass of the RCC3 is 1141.8886, the same as the calculated m/z of the protonated dried RCC3 1141.8892. The mass spectra for the RuNCs was not achieved because of the low intensities of the expected large number of isotope peaks due to the broad and continuous small particle size distribution. Related to Figures 1 and 2.

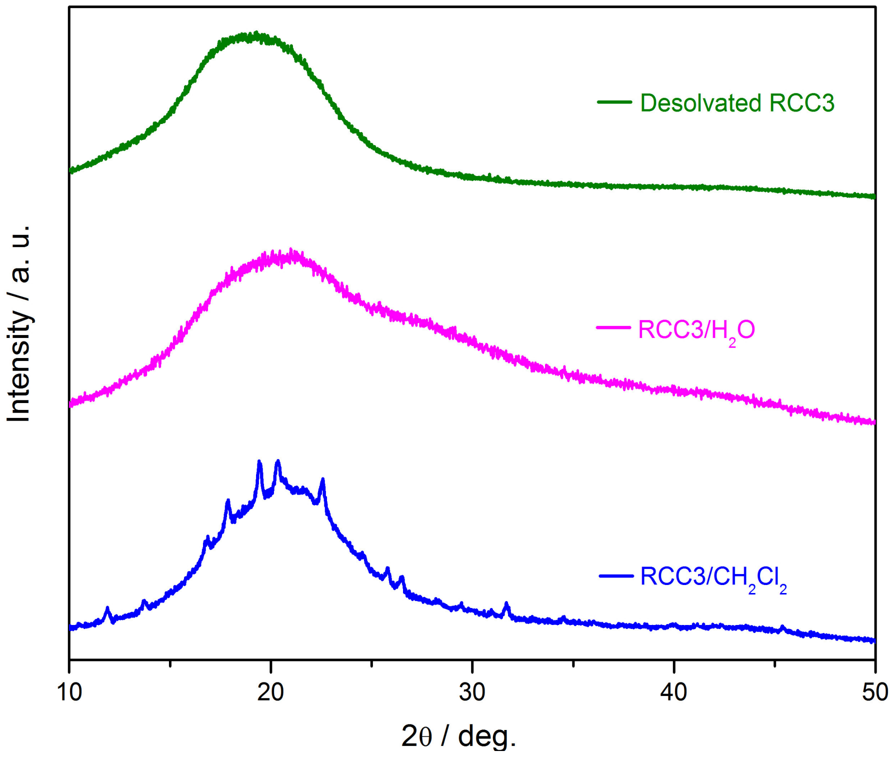

Figure S6. XRD patterns of RCC3 cage with CH_2Cl_2, H_2O, and the desolvated RCC3. As shown in these patterns, the desolvated RCC3 shows a broad band in the range of 10–30° because of the collapse of the cavities upon solvent removal. Surprisingly, the diffraction peaks of the crystalline RCC3 reappear with the addition of CH_2Cl_2 to desolvated RCC3, while such phenomenon is not observed with the addition of water, indicating that the collapsed cavities of RCC3 have great affinity to hydrophobic molecules returning to the original crystalline state. This feature helps the incorporation of metal precursors into cage cavities by the reverse double-solvents approach. Related to Figure 2.

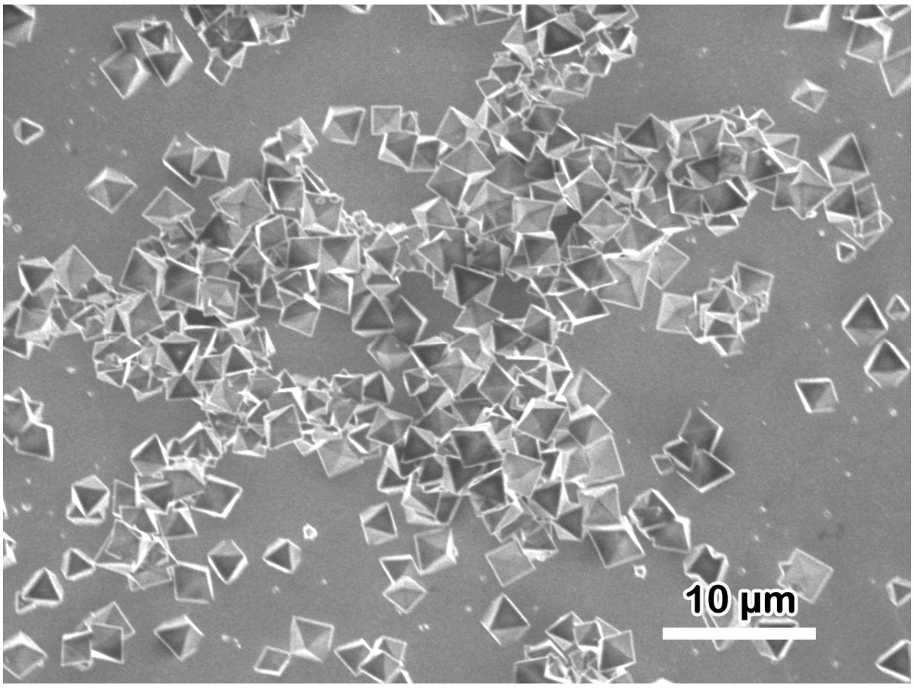

Figure S7. FESEM image of RCC3 (with purification). Related to Figure 2.

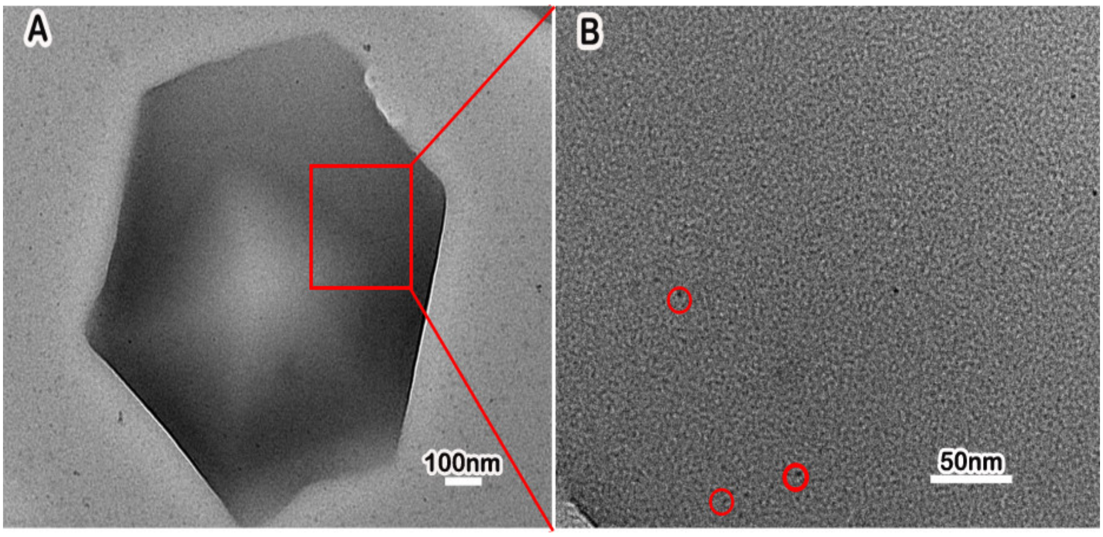

Figure S8. (**A**) TEM of RuNCs@RCC3 with the morphology of octahedron. (**B**) Enlarged TEM image of RuNCs@RCC3. From the TEM image, the Ru nanoparticles were hardly observed on the surface of RCC3 due to its ultrafine size with Ru nanoclusters inside the cage cavities. Related to Figure 2.

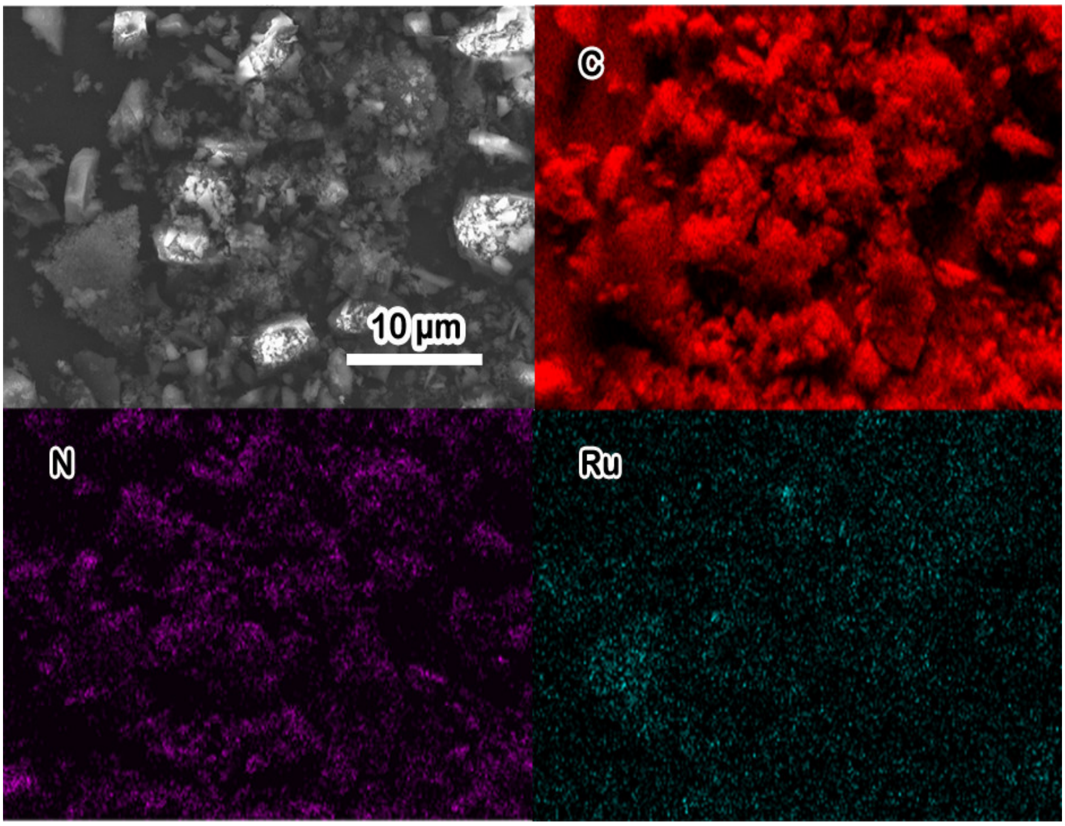

Figure S9. FESEM image for EDS mapping of RuNCs@RCC3. According to Supplemental Figure 9, the C and N elements are the typical distribution while the Ru element is rare on the surface of RCC3, which is consistent with the TEM image. Also, countless catalytic sites are beneficial to promote the formation/decomposition of Li_2O_2 in the electrolyte during the cycling of Li-O_2 battery, which provides a nice electrochemical performance improvement of Li-O_2 battery with RuNCs/CNT cathodes. Related to Figure 2.

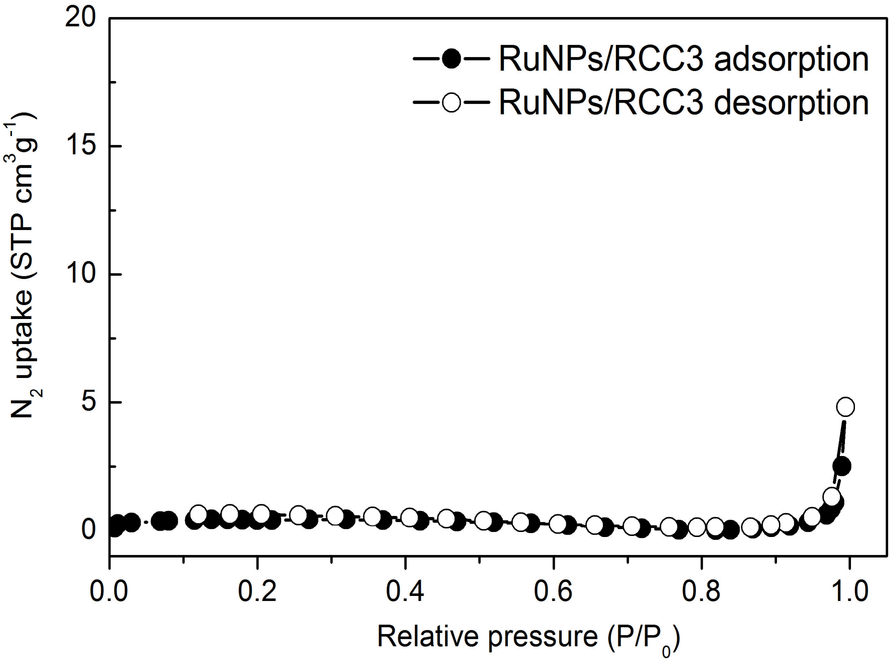

Figure S10. N_2 adsorption/desorption isotherms. N_2 adsorption/desorption isotherms of RuNPs/RCC3. As revealed, the RuNPs/RCC3 shows nonporous characteristics with a low surface area of 2.98 m^2 g^{-1}, which should be ascribed to the aggregation of RuNPs on the surface of RCC3. Related to Figure 2.

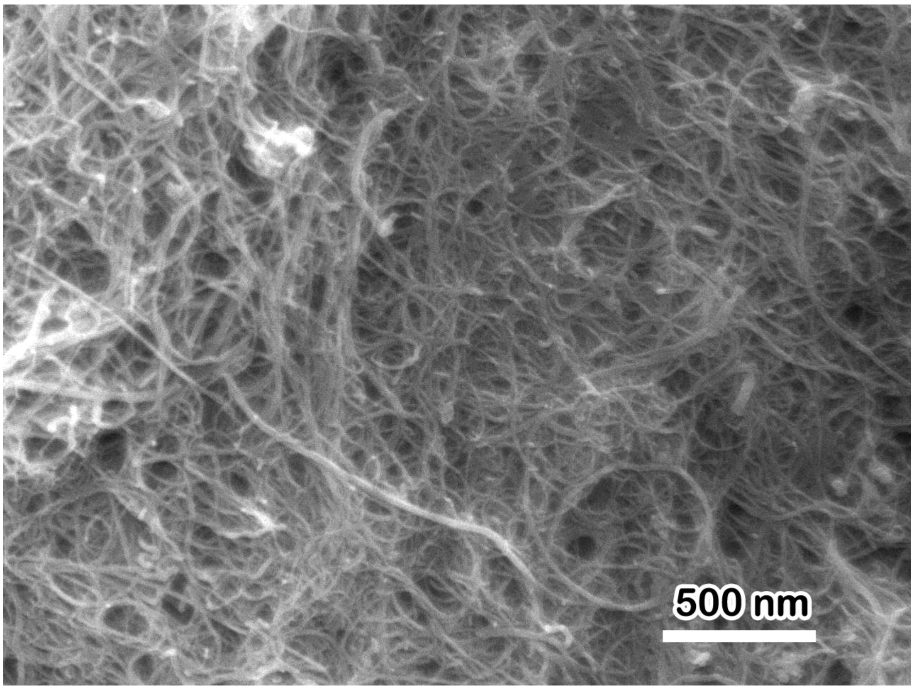

Figure S11. FESEM image of the pristine CNT cathode. To illustrate the role of RuNCs@RCC3 in the morphological evolution of the discharge products during discharge and charge process, CNT is selected as the cathode in the Li-O_2 cells. As revealed by the FESEM results, the superior pore structure interconnected through CNT can be a key condition for tailoring the growth and the morphology of Li_2O_2. Related to Figure 3.

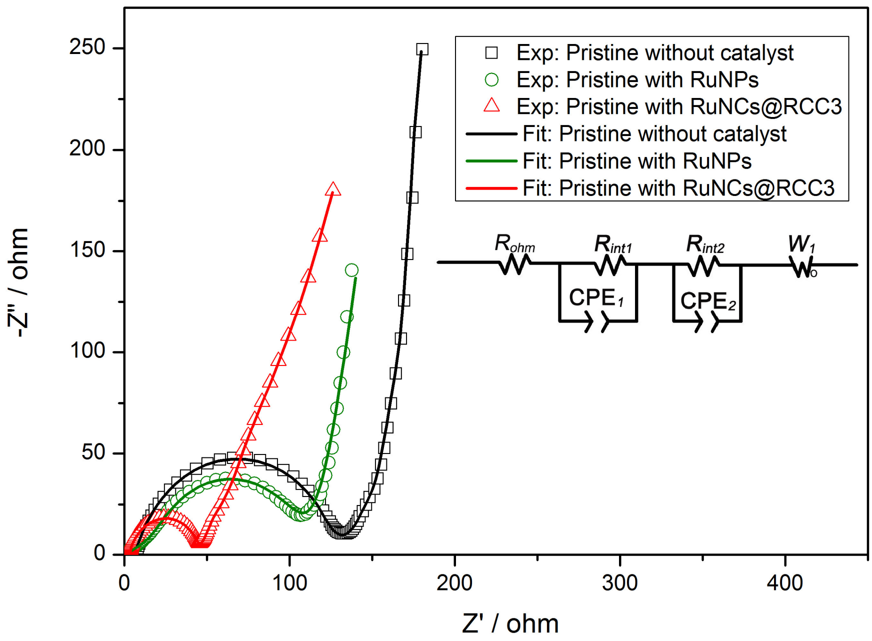

Figure S12. Electrochemical impedance spectra of the Li–O_2 cells with the CNT cathodes without catalyst, with RuNPs and with RuNCs@RCC3 at pristine stage. The equivalent-circuit parameters are obtained by curve fitting using the Zview software with the equivalent circuit shown in the inset. The data points are the measured values and the solid line is the calculated impedance curve using the equivalent circuit. R_{ohm} is the Ohmic resistance of the Li-O_2 cell. R_{int1} is the interfacial resistance between the Li anode and the electrolyte. R_{int2} is the interfacial resistance between the cathode and the electrolyte. W_1 is the Warburg impedance arising from the Li^+ concentration gradients. The fitting values of R_{int2} are 336, 38.56, and 28.57 Ω cm^{-2} for the CNT cathodes without catalyst, with RuNPs, with RuNCs@RCC3, respectively. Related to Figure 3.

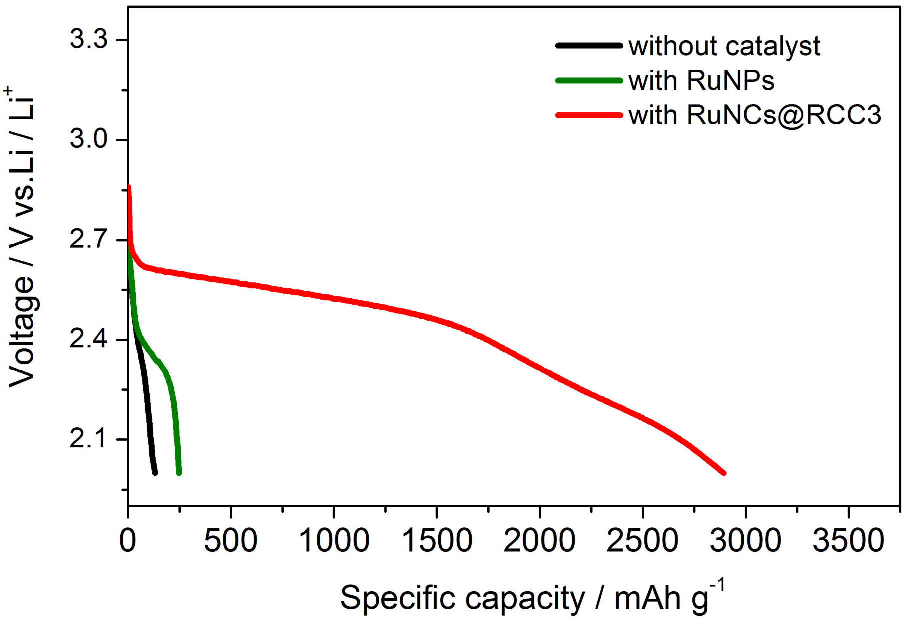

Figure S13. Rate capability measurement. Galvanostatic discharge curves of the Li-O_2 cells with the CNT cathodes without catalyst, with RuNPs, and with RuNCs@RCC3 at the current density of 1000 mA g^{-1}. Related to Figure 3.

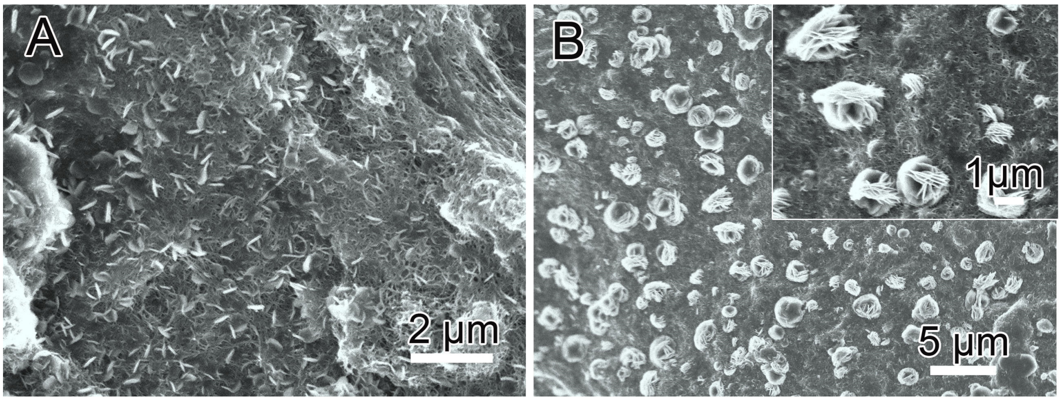

Figure S14. (**A**) FESEM image of the CNT cathode without catalyst after being discharged at a current density of 200 mA g^{-1} and a specific capacity of 500 mAh g^{-1}. (**B**) FESEM image of image the CNT cathode with RuNCs@RCC3 after being discharged at a current density of 200 mA g^{-1} and a specific capacity of 500 mAh g^{-1}. At the initial stage, the discharged product on the CNT cathode without catalyst shows small discs (100–200 nm in size) morphology, which is consistent with the reported studies (Xu et al., 2016). In sharp contrast, aggregated micrometer-sized flower-like products appear on the CNT cathode with RuNCs@RCC3. This obvious difference can be ascribed to the growth pathway of Li_2O_2 with RuNCs@RCC3 possessing more reaction sites which can induce more LiO_2* nucleation to generate large Li_2O_2. Related to Figure 3.

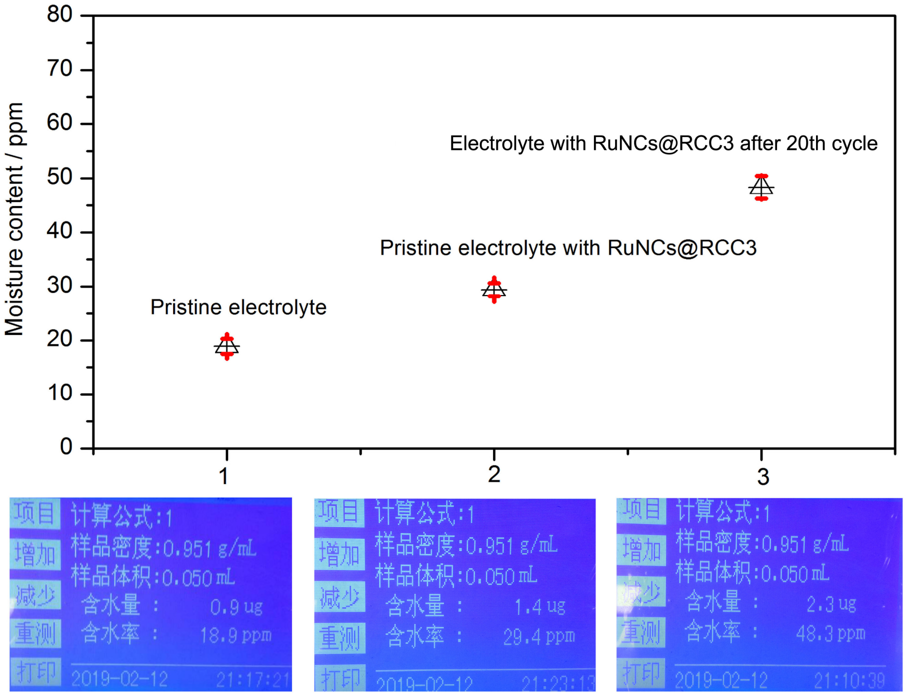

Figure S15. The related data about Karl Fischer (KF) titration of the pristine electrolyte, the pristine electrolyte with RuNCs@RCC3 and after 20th cycle of the electrolyte with the RuNCs@RCC3 catalyst, the red line represents the error relative to the average value. The corresponding resulting pictures by the instrument are also displayed. The results demonstrate that the water content in the electrolyte shows slight increment with the addition of RuNCs@RCC3. Even after the 20th cycle, the water content in electrolyte with RuNCs@RCC3 is still lower than 50 ppm. Related to Figure 3.

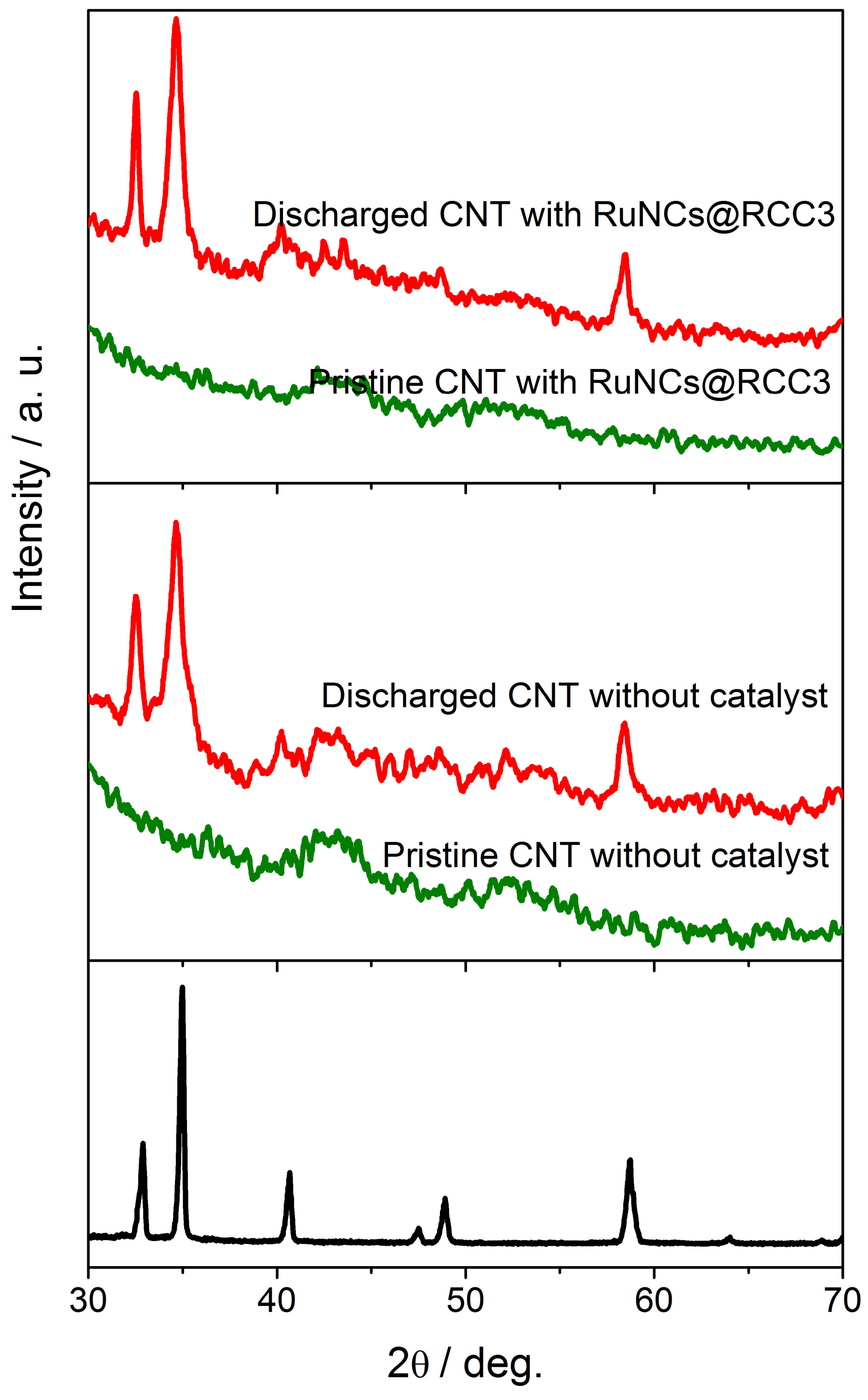

Figure S16. The XRD patterns of the discharged pristine CNT cathode without catalyst and the CNT cathode with RuNCs@RCC3. The spectra for standard Li_2O_2 are also shown for reference. The XRD peaks of the discharged cathodes can be assigned to Li_2O_2, although the morphologies of the discharge products are different. Related to Figure 3.

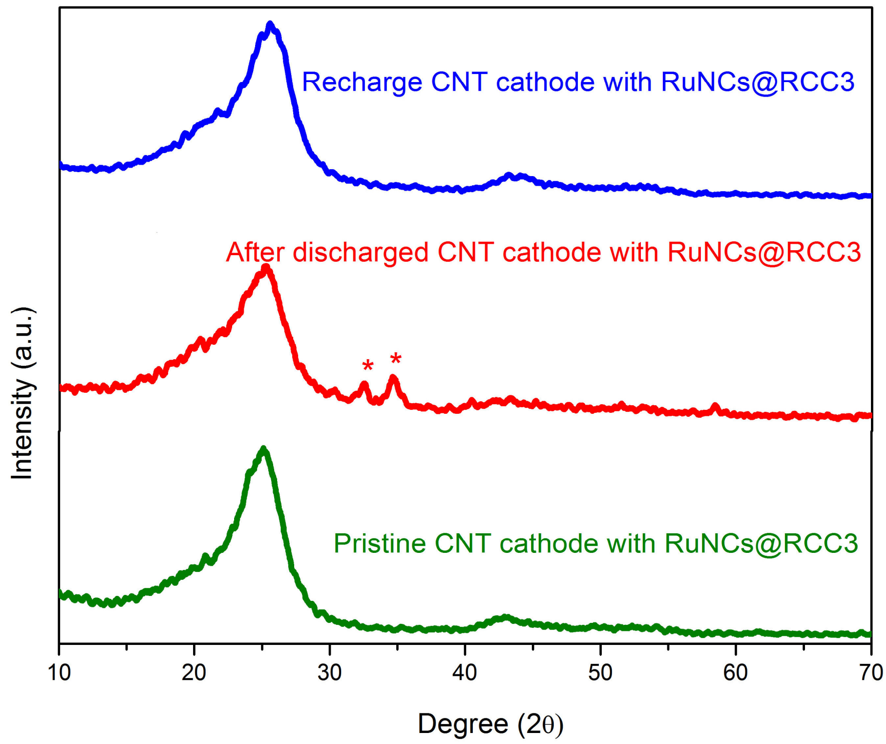

Figure S17. The XRD patterns of the pristine, discharged and recharged CNT cathode with RuNCs@RCC3 which is perfomed down to 2-Theta angles of 10 degrees. Related to Figure 3.

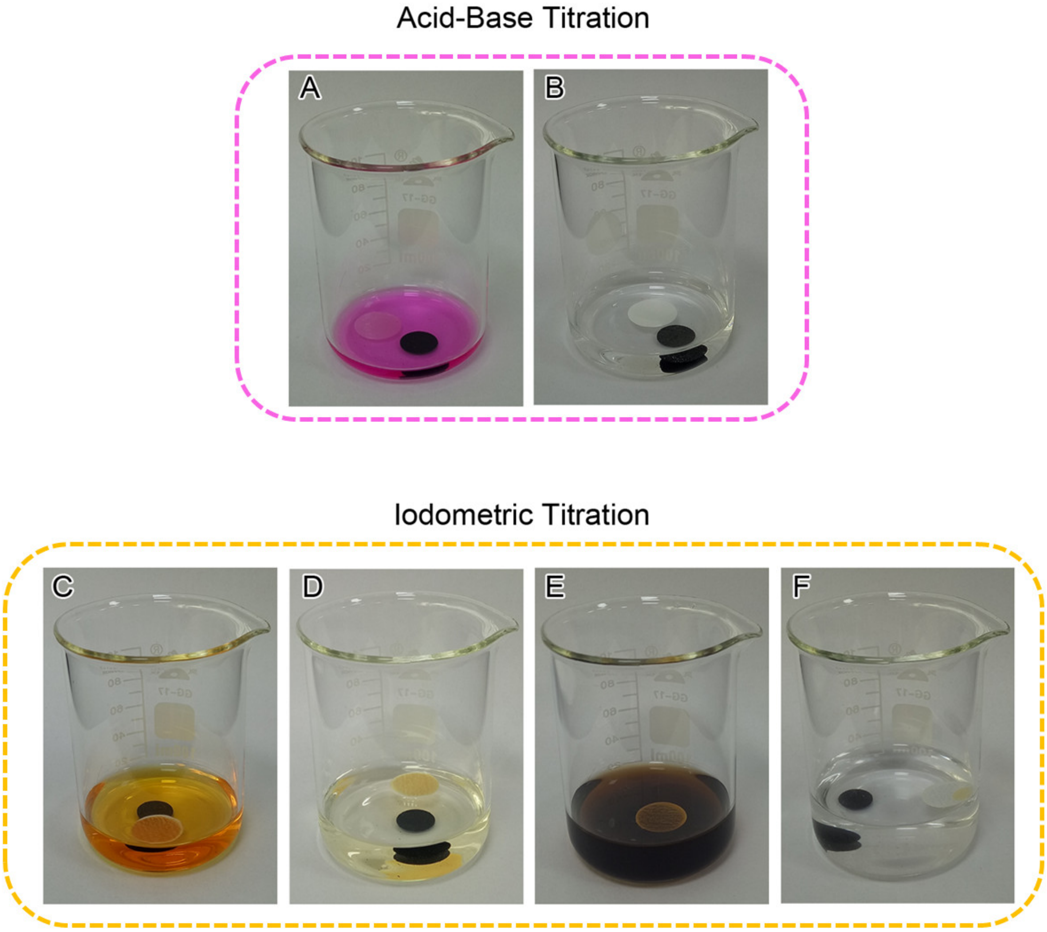

Figure S18. Color changes during both acid-base titration and iodometric titration. Related to Figure 3.

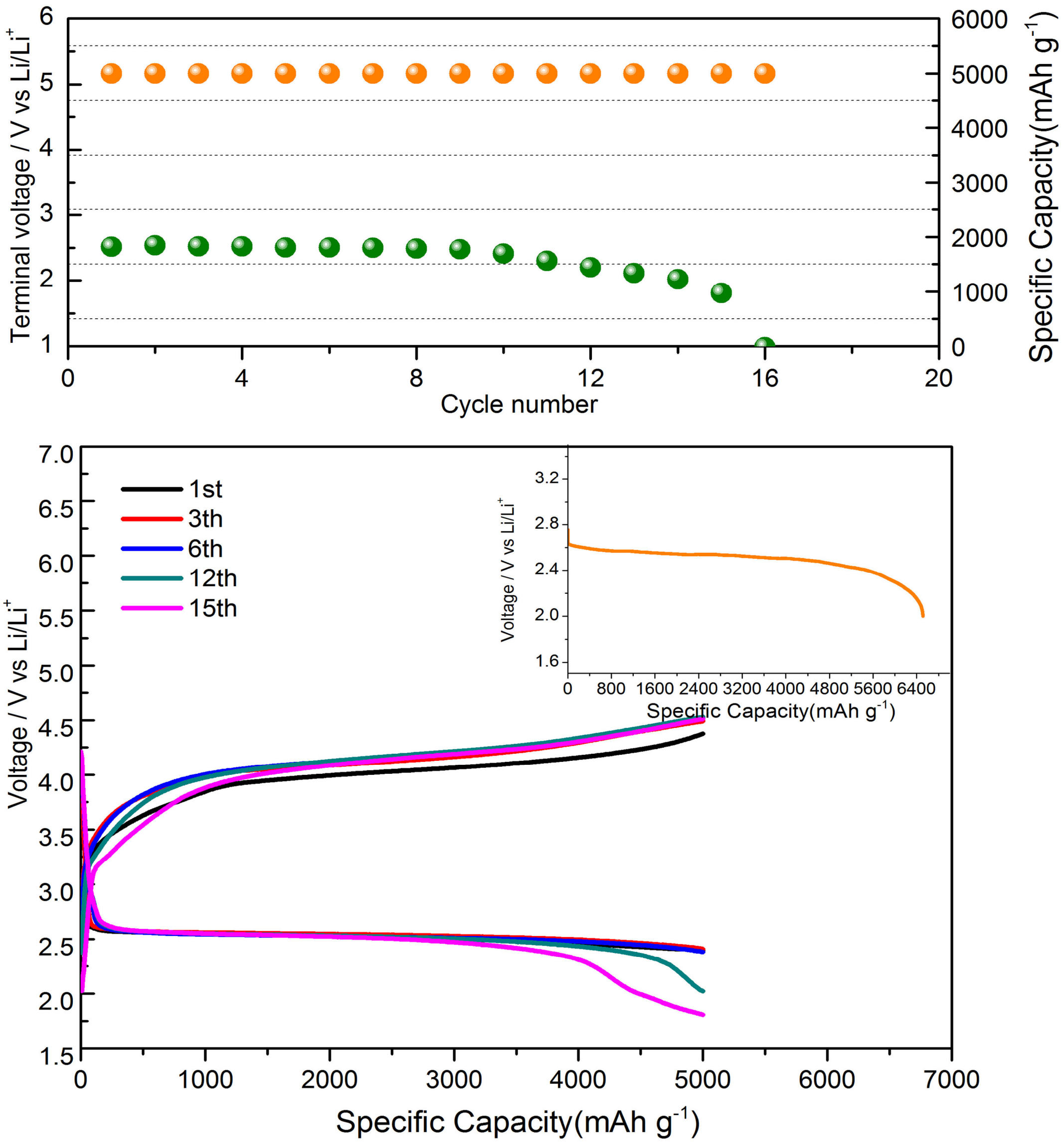

Figure S19. Electrochemical performance. Variation of voltage on the terminal of discharge of the Li-O_2 cells at a current density of 500 mA g^{-1} and a specific capacity limit of 5000 mAh g^{-1} with RuNCs@RCC3 catalyst. The inset represents the galvanostatic discharge curve of the Li-O_2 cells with RuNCs@RCC3. Related to Figure 4.

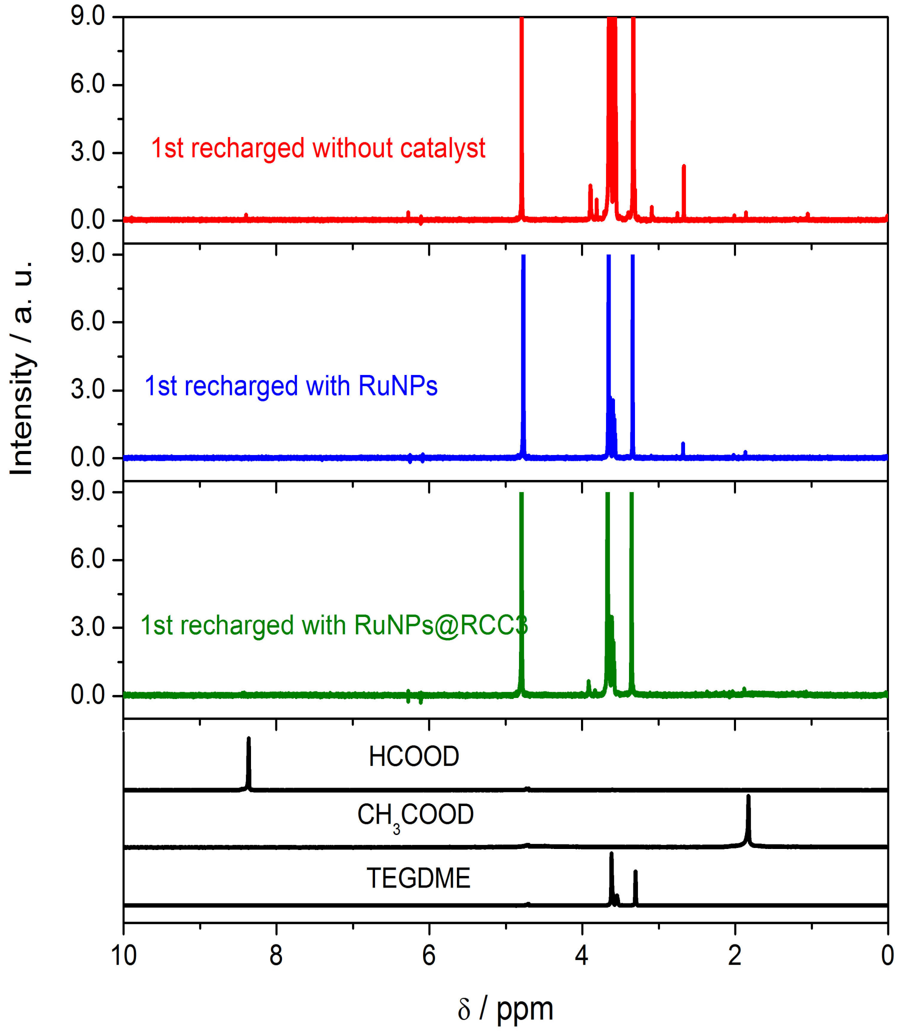

Figure S20. ^{1}H NMR spectra of the pristine CNT cathodes without catalyst, with RuNPs and RuNCs@RCC3 after the first discharged. The spectra of TEGDME (tetraethylene glycol dimethyl ether), HCO_2Li, and CH_3CO_2Li are also shown for reference. NMR measurement further demonstrates that there are no byproducts observed for the discharged products of the CNT cathode with RuNCs@RCC3, while irreversible discharge products accumulated on the other two cathodes during cycling. Related to Figure 5.

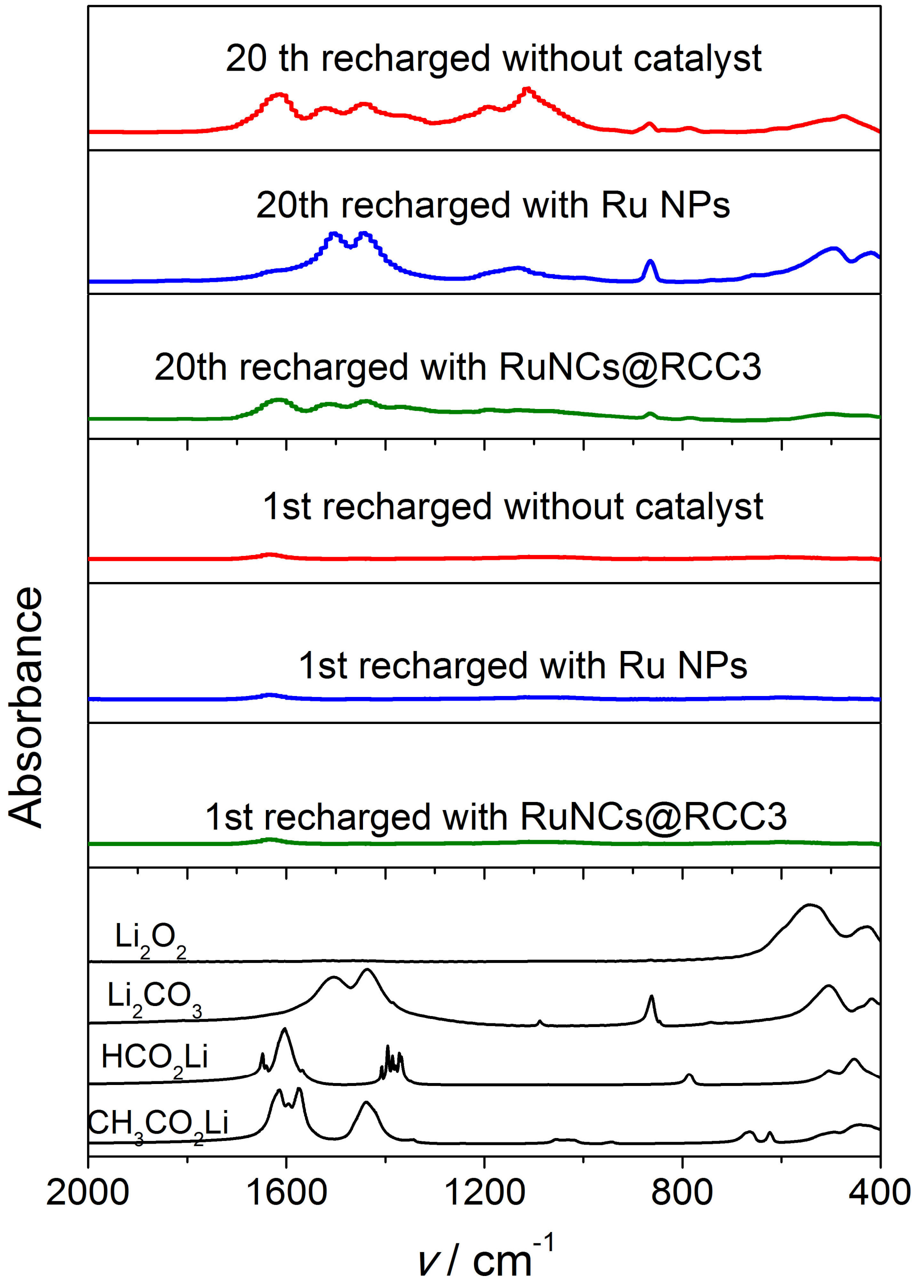

Figure S21. FTIR spectra of the CNT cathode after different discharge–charge cycles with RuNCs@RCC3, RuNPs and without catalyst. The spectra for Li_2O_2, Li_2CO_3, HCO_2Li and CH_3CO_2Li are also shown for reference. Related to Figure 5.

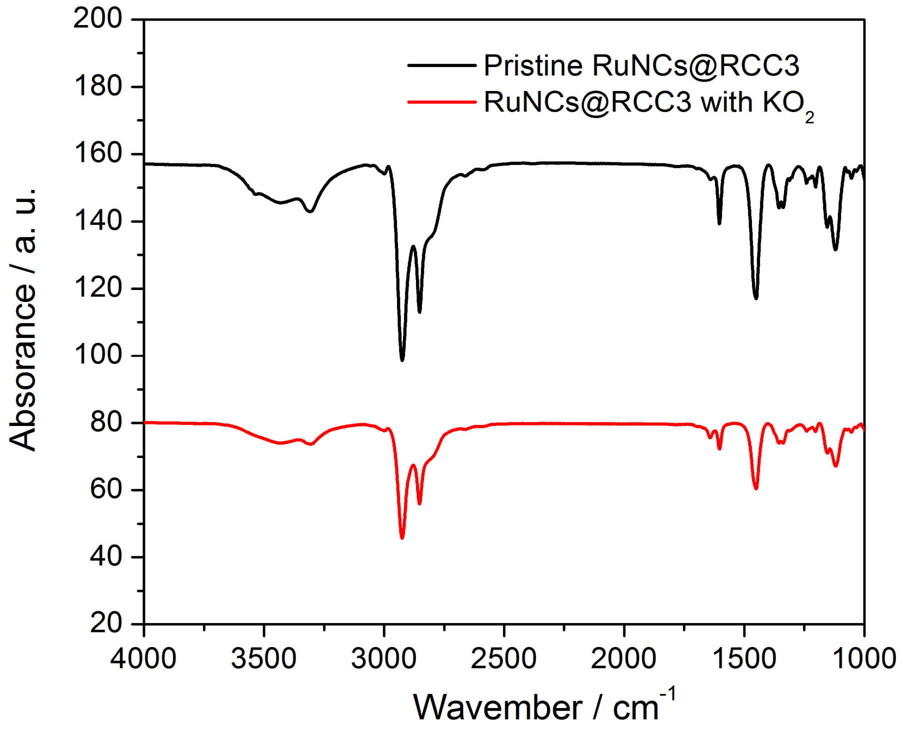

Figure S22. FTIR spectra of RuNCs@RCC3 and RuNCs@RCC3 with KO_2. We generated metastable superoxide from the well-known reaction of KO_2 with dicyclohexyl-18-crown-6 (crown ether) and DMSO mixed solutions, which complexes the K^+ to metathesis with a lithium salt, forming metastable solvated LiO_2(Black et al., 2012). These results clearly demonstrate that the RuNCs@RCC3 with KO_2 has nearly the same structure as the pristine RuNCs@RCC3, showing the superior stability of RuNCs@RCC3 towards the LiO_2. Related to Figure 5.

Supplemental Tables

Table S1. Atomic mass ratios of C, H and N in RCC3. Related to Figure 2.

Run details		Results		
Run	Weight (mg)	C (wt.%)	H (wt.%)	N (wt.%)
1	2.8040	72.04	9.310	13.75
2	2.8620	73.58	9.351	14.07
3	2.3270	71.78	9.310	13.62
Theoretical value		75.72	9.47	14.72

Table S2. Results of acid-base titration and iodometric titration. The titration is conducted on cathodes (with separator) to quantitatively analyze the amount of both deposited products and soluble products during discharge. Related to Figure 3.

cathode	Specimen	5mM $Na_2S_2O_3$ dosage(mL)	$nLi_2O_{2,t}$ (μmol)	$nLi_2O_{2,e}$ (μmol,1.5mAh)	Y_{Li2O2}(%) Mean value
Without catalyst	1	7.92	19.80	27.99	71.4±1.4
	2	8.10	20.25	27.99	
	3	7.98	19.95	27.99	
With RuNPs	1	7.28	18.20	27.99	64.0±1.6
	2	7.16	17.90	27.99	
	3	6.98	17.65	27.99	
With RuNCs@RCC3	1	8.32	20.80	27.99	73.5±1.3
	2	8.20	20.50	27.99	
	3	8.16	20.40	27.99	

Where $nLi_2O_{2,t}$ is the titrated amount of Li_2O_2, and $nLi_2O_{2,e}$ is the expected amount of Li_2O_2 given a 2.000 e^-/Li_2O_2 process during discharge (18.66 μmols Li_2O_2 per mAh would be expected to give a 2 e^-/Li_2O_2 process). The obtained values are calculated with standard deviation of three replicate trials. Li_2O_2 yields (Y_{Li2O2}) are calculated using the following equation:

$Y_{Li2O2}= nLi_2O_{2,t}/nLi_2O_{2,e}*100\%$

Transparent Methods

Chemicals and Materials: All chemicals were directly used without further purification. Methanol, dichloromethane, (R,R)-1,2-diaminocyclohexane, trifluoroacetic acid, sodium borohydride, Ruthenium (III) acetylacetonate, Ruthenium chloride hydrate, Starch soluble, Sodium Thiosulfate pentahydrate, Potassium iodide, anolyte for coulometric Karl Fischer titration and Ammonium Nitrate were purchased from Aladdin Reagent, 1,3,5-triformylbenzene was purchased from Zhengzhou alpha chemical co. LTD, Ammoniun Molybdate was purchased from 9 Ding Chemistry, Phenolphthalein was purchased from BeiJing Chemistry, Li_2CO_3, CH_3CO_2Li and HCO_2Li were purchased from Sigma-Aldrich. Li_2O_2 was purchased from Acros. CNT was purchased from Cnano Technology Limited Company. Lithiated Nafion was purchased from the DuPont Company. CP (carbon paper) was purchased from Torray. Diethylamine, ethanol, N-methyl-2-pyrrolidinone, TEGDME, LiTFSI and deuterium oxide, and deuterochloroform were purchased from Aladdin Reagent.

Synthesis of RuNCs@RCC3 by a reverse double-solvents approach: The synthesis method for the RuNCs@RCC3 was described by Yang et al. (2018) with a slight modification. In a typical synthesis, 85 mg of dried RCC3 powder was dispersed in 20 mL water and sonicated for 20 min. Then 0.02 mL $Ru(C_5H_7O_2)_3/CH_2Cl_2$ containing 0.01 mmol Ru^{3+} solution as the hydrophobic solution was slowly added into the cage/water dispersion, and the resulting mixture was continuously stirred for 3 h. Finally, 0.5 mL of the newly prepared $NaBH_4$ (50 mg) aqueous solution was quickly added into the above mixture and kept stirring for 3 h. The obtained samples were centrifuged and washed with water and DMF, then resolved in CH_2Cl_2 subsequent with removing the solvents. The obtained solid samples were dried in air at 70 °C for 2.5 h and further used for the catalytic reactions.

Synthesis of CC3R: 10 mL CH_2Cl_2 was slowly added to 0.5 g 1,3,5-triformylbenzene labeled as A, and the (R,R)-1,2- diaminocyclohexane (0.5 g, 4.464 mmol) in the same solvent labeled as B .Then the B solution was slowly added into the A solution. Finally, 10 μL trifluoroacetic acid was directly added into the above solution for imine bond formation. The mixture solution was capped and kept for one week for successful crystallization on the edges of the vessel. Subsequently, the crystalline product was centrifuged and washed with CH_2Cl_2/CH_3OH mixture (v/v, 5/95), dried at 100 °C under vacuum overnight.

Synthesis of RCC3: The *as-prepared* CC3R was completely dissolved in a 25 mL CH_2Cl_2/CH_3OH mixture (v/v,1/1), and 0.5 g $NaBH_4$ was added into the mixture and stirred for 15 h at room temperature followed by the addition of 1 mL distilled water, stirred for 9 h. Then the solvent was removed via rotary evaluation, washed with water and centrifuged, the final product was dried at 70 °C under vacuum overnight.

Purification of RCC3 cages: 100 mg RCC3 was dissolved in 10 mL acetone for 24 h followed by the centrifugation. The obtained product could be completely dissolved in 10 mL CH_2Cl_2/CH_3OH mixture (v/v, 1/1) by constant stirring. 0.1 mL distilled water was added and stirred for 48 h. The solvent was removed and the pure RCC3 was obtained.

Synthesis of the RuNPs-Free catalyst: The RuNPs-Free catalyst was prepared by a similar process to the Ru-NPs-Free catalyst, except for unused RCC3.

Synthesis of the RuNPs/RCC3 catalyst: The RuNPs/RCC3 catalyst was synthesized by the conventional impregnation method. 85 mg of RCC3 powder was ultrasonically dissolved in 30 mL H_2O, stirred for 20 min. Then $RuCl_3.xH_2O$ (0.01 mmol) in 0.02 mL CH_3OH was subsequently added and stirred for 20 min

followed by the addition of 50 mg $NaBH_4$, stirred for 3 h, resulting in the generation of RuNPs/RCC3 catalyst.

Electrolytes: Lithium bis(trifluoromethane sulfonyl)imide (LiTFSI) was the preferred lithium salt for all experiments reported in this manuscript. 1 M LiTFSI in tetraethylene glycol dimethylether (TEGDME) was prepared. The water content in electrolyte is measured by KarlFischer titration.

Preparation of RuNCs@RCC3 electrolyte: 20 mg RuNCs@RCC3 catalyst was directly immersed into 1 mL CH_2Cl_2 with 2 mL TEGDME (1 M LiTFSI) and stirred for 2 days.

Preparation of RuNPs/CNT cathode: The synthesis method of RuNPs was similar to the RuNCs@RCC3 without using RCC3. Then the solid powder was mixed with 3 mL CH_2Cl_2/TEGDME (1 M LiTFSI) for comparison.

Li-O_2 cell preparation and electrochemical measurements: The electrochemical performance of Li-O_2 cell was tested in a 2025-type coin cell. All of the cells were assembled in a glove box under an Ar atmosphere with a lithium metal foil anode, a glass fiber separator, an oxygen cathode and an electrolyte containing 1 M LiTFSI in TEGDME. The pristine CNT cathode was prepared by coating homogenous ink composed of a mixture of 80 wt% CNT powder and 20 wt% Poly(vinylidene fluoride) onto the CP current collector. And 30 wt% RuNPs, 50 wt% CNT powder and 20 wt% Poly(vinylidene fluoride) deposited on CNT cathode was prepared in the same manner. The active material loading was 0.45 mg cm^{-2}. The electrochemical performances of the CNT cathodes with RuNCs@RCC3, with RuNPs, and without catalyst were tested in a specific capacity-controlled mode under various current densities. The electrochemical impedance spectroscopy of the cell was evaluated using a CHI660E electrochemical workstation within a frequency range of 10^5 to 10^{-2} Hz.

Characterization: The morphology and structures of the materials were characterized using various physiochemical techniques, including X-ray diffraction (XRD), ^{1}H and ^{13}C nuclear magnetic resonance (NMR) spectroscopy, Fourier transform infrared spectra (FTIR), mass spectrometry, field emission scanning electron microscopy (FESEM), and transmission electron microscopy (TEM). The discharge and recharge products were characterized using XRD, SEM, NMR technology.

Sample preparation procedure for NMR measurements: The side reactions of the recharged cathode were analyzed using NMR measurements. 2025-type coin cells were disassembled in the Ar-filled glove box (H_2O level < 0.1 ppm, O_2 level < 0.1 ppm). The cathode was rinsed with pure anhydrous acetonitrile for several times to remove residual salts and/or ionic liquids, followed by a rinse with Deuterated D_2O (99.8%) and immediately transferred into the Nuclear magnetic tube for testing.

Chemical Titrations: The chemical titration processes performed in this study according to the previous study (McCloskey, et al., 2013; Qiao, et al., 2017). Here, a brief description of the procedures has been displayed. To collect the full information of soluble products, the obtained cathodes and glass fiber separators of cells without catalyst and with RuNPs were directly used without washing and evaporating procedures. Yet the cathode and glass fiber separator of the cell with RuNCs@RCC3 was immersed into CH_2Cl_2 for 5 min so as to getting rid of Ru, because Ru would catalyze the decomposition reaction of hydrogen peroxide and then caused low Li_2O_2 yields. Then the sample was taken out of the glove box, and put into a conical flask with 5.0 mL ultrapure DI-water immediately. The flask was vigorously shaken for 20 s to promote the complete reaction of Li_2O_2 with H_2O. The involved reaction is as follows:

$$Li_2O_2 + 2H_2O = 2LiOH + H_2O_2$$

The whole titration process can be divided into two steps: (1) Acid-Base Titration (towards OH^-) and (2) Iodometric Titration (towards H_2O_2). For the acid-base titration, the base was titrated using a standardized 5 mM HCl solution, with the end point indicated by a drop of phenolphthalein in isopropanol. The involved reaction is as follows:

$LiOH + HCl = LiCl + H_2O$

The iodometric titration was straightly followed with the addition of three reagents into the existed solution in sequence: 1 mL 2wt% KI aqueous solution, 1 mL 3.5 M H_2SO_4 solution and 50 μL Mo-based catalyst solution. The Mo-based catalyst solution was prepared by dissolving 0.5 g ammonium molybdate ($(NH_4)_2MoO_4$) and 1.5 g ammonium nitrate (NH_4NO_3) into 5 mL 30 wt% ammonia aqueous solution, then diluting to 25 mL using ultrapure DI-water. The resultant solution turned to an orange color due to the formation of I_2. Then, the I_2 was titrated to a faint pale yellow color by employing 5 mM $Na_2S_2O_3$ aqueous solution. 0.5 mL 1% starch solution was added for the precise end-point detection. The solution rapidly turned to a dark blue color and the titration was resumed till the color was completely disappeared. The involved reaction is as follows:

$H_2O_2 + 2KI + H_2SO_4 = I_2 + K_2SO_4 + 2H_2O$

$I_2 + 2Na_2S_2O_3 = Na_2S_4O_6 + 2NaI$

The details of analysis on original data and images of color-changes are shown in the corresponding Supplementary Information Section above (Figure S18 and Table S2).

Supplemental References

Dong, Q., Yao, X., Zhao, Y., Qi, M., Zhang, X., Sun, H., He, Y., and Wang, D. (2018). Cathodically Stable Li-O_2 Battery Operations Using Water-in-Salt Electrolyte. Chem 4, 1345-1358.

McCloskey, B.D., Valery, A., Luntz, A. C., Gowda, S. R., Wallraff, G. M., Garcia, J. M., Mori,T., and Krupp, L. E. (2013). J. Phys. Chem. Lett. 4, 2989-2993.

Qiao, Y., Wu, S., Yi, J., Sun, Y., Guo, S., Yang, S., He, P., and Zhou, H. (2017). From O_2^- to HO_2^-: Reducing by-products and overpotential in Li-O_2 batteries by water addition. Angew. Chem. Int. Ed. 56, 4960-4964.

Qiao, Y., He, Y., Wu, S., Jiang, K., Li, X., Guo, S., He, P. and Zhou, H. (2018). MOF-Based Separator in an Li−O_2 Battery: An Effective Strategy to Restrain the Shuttling of Dual Redox Mediators. ACS Energy Lett. *3*, 463-468.

Zhai, D., Wang, H.-H., Yang, J., Lau, K. C., Li, K., Amine, K., and Curtiss L. A. (2013). Disproportionation in Li-O_2 batteries based on a large surface area carbon cathode. J. Am. Chem. Soc. *135*, 15364-15372.
